# Identification, Functional Characterization and Regulon Prediction of a Novel Two Component System Comprising BAS0540-BAS0541 of *Bacillus anthracis*

**DOI:** 10.1371/journal.pone.0158895

**Published:** 2016-07-08

**Authors:** Monisha Gopalani, Alisha Dhiman, Amit Rahi, Divya Kandari, Rakesh Bhatnagar

**Affiliations:** Laboratory of Molecular Biology and Genetic Engineering, School of Biotechnology, Jawaharlal Nehru University, New Delhi-110067, India; Universidad de Costa Rica, COSTA RICA

## Abstract

Two component systems (TCSs) can be envisaged as complex molecular devices that help the bacteria to sense its environment and respond aptly. 41 TCSs are predicted in *Bacillus anthracis*, a potential bioterrorism agent, of which only four have been studied so far. Thus, the intricate signaling network contributed by TCSs remains largely unmapped in *B*. *anthracis* and needs comprehensive exploration. In this study, we functionally characterized one such system composed of BAS0540 (Response regulator) and BAS0541 (Histidine kinase). BAS0540-BAS0541, the closest homolog of CiaRH of *Streptococcus* in *B*. *anthracis*, forms a functional TCS with BAS0541 displaying autophosphorylation and subsequent phosphotransfer to BAS0540. BAS0540 was also found to accept phosphate from physiologically relevant small molecule phosphodonors like acetyl phosphate and carbamoyl phosphate. Results of qRT-PCR and immunoblotting demonstrated that BAS0540 exhibits a constitutive expression throughout the growth of *B*. *anthracis*. Regulon prediction for BAS0540 *in B*. *anthracis* was done *in silico* using the consensus DNA binding sequence of CiaR of *Streptococcus*. The predicted regulon of BAS0540 comprised of 23 genes, which could be classified into 8 functionally diverse categories. None of the proven virulence factors were a part of the predicted regulon, an observation contrasting with the regulon of CiaRH in *Streptococci*. Electrophoretic mobility shift assay was used to show direct binding of purified BAS0540 to the upstream regions of 5 putative regulon candidates- BAS0540 gene itself; a gene predicted to encode cell division protein FtsA; a self–immunity gene; a RND family transporter gene and a gene encoding stress (heat) responsive protein. A significant enhancement in the DNA binding ability of BAS0540 was observed upon phosphorylation. Overexpression of response regulator BAS0540 in *B*. *anthracis* led to a prodigious increase of ~6 folds in the cell length, thereby conferring it a filamentous phenotype. Furthermore, the sporulation titer of the pathogen also decreased markedly by ~16 folds. Thus, this study characterizes a novel TCS of *B*. *anthracis* and elucidates its role in two of the most important physiological processes of the pathogen: cell division and sporulation.

## Introduction

Successful adaptation and survival of bacteria depends on their explicit ability to sense environmental inconstancies. Two component systems (TCSs) are a form of stimulus-response couplers found ubiquitously in bacteria, which are ingeniously exploited for environmental information processing. An archetypal TCS is comprised of a membrane bound stimulus sensor: the Histidine Kinase (HK), and a cytosolic responsive component: the Response Regulator (RR) [1]. The HK houses an N-terminal sensing domain and a conserved C-terminal catalytic center consisting of the DHp (dimerization and histidine phosphotransfer) and CA (catalytic and ATP-binding) domains. While the DHp domain is the site for autophosphorylation, dimerization and phosphatase activity in bifunctional kinases, the CA domain binds to ATP required for HK autophosphorylation [2]. The RR consists of a structurally conserved N-terminal receiver domain and a C-terminal effector domain. The latter in majority of the cases is a DNA binding domain responsible for bringing about changes in the transcriptional program of the cell [3]. Upon sensing a signal, the HK gets autophosphorylated on a conserved histidine residue in the DHp domain. This phosphate is then transferred to the conserved aspartate residue of its cognate RR. Thus, the RR becomes activated and executes an adaptive response by binding to the upstream regulatory regions of genes that constitute its regulon. This two-step phosphotransfer constitutes the basic scheme of TCSs [4]. A TCS can govern multifarious processes like membrane fluidity, metabolic pathways, sporulation, motility, biofilm formation, growth and viability and so on [4–6]. Moreover, in many pathogens, expression of classical virulence factors and host- pathogen interactions are also regulated by TCSs.

*Bacillus anthracis* is a Gram-positive, spore-forming bacterium responsible for the fatal disease anthrax, predominantly a zoonotic disease affecting herbivores and domestic animals. However, occasionally humans can acquire the disease after contact with infected animals or contaminated animal products [7]. The fact that its spores can remain viable for decades and can be easily disseminated as aerosols makes it a biological warfare agent as exemplified by the episode of *B*. *anthracis* spore attacks through the US mail system after September 11, 2001 [8]. The key virulence determinants of the pathogen are plasmid encoded and include a poly gamma d-glutamic acid capsule, which helps the pathogen in evading phagocytosis inside the host, along with a tripartite toxin [9].

Environmental sensing is indispensable for maintaining the dual lifestyle of *B*. *anthracis*, which in part may be executed by TCSs. The complexity of these signaling systems largely depends on the lifestyle and gamut of environmental fluctuations that an organism is exposed to during its life cycle [10–12]. Conceptually, bacteria that specialize as pathogens are often exposed to relatively stable host environments as compared to environmental bacteria, which reside in diverse habitats. This might be the reason for a relatively low number of TCSs in many pathogens. While two pathogens closely related to *B*. *anthracis*, *Streptococcus pneumoniae* (13 TCSs) [13] and *Staphylococcus aureus* (17 TCSs) [14] perfectly fit into this argument, *B*. *anthracis* clearly stands out having an exceptionally high number of TCSs. There are 52 HKs and 51 RRs predicted in *B*. *anthracis*, out of which 41 exist as HK-RR pairs [15]. However, because of the limited research on TCSs of the *Bacillus cereus* group (to which *B*. *anthracis* also belongs) done till date, these have been subjected to marginal scrutinization, leaving them largely unexplored. As a result of which, only 4 of the 41 TCSs present in *B*. *anthracis* have been characterized till date.

The contribution of TCSs in the maintenance of a specialized lifestyle pattern, infection, host adaptation, and pathogenesis of *B*. *anthracis* needs to be reconnoitered, which in turn would require inquisitive study of these systems from scratch and our present study aims at doing the same. In this study, we demonstrate that BAS0540-BAS0541 constitutes a classical TCS of *B*. *anthracis*. The RR and HK encoded by BAS0540 and BAS0541, respectively, follow the conventional paradigm of the modular nature of TCS proteins and their operonic organization. BAS0541 exhibited autophosphorylation and phosphotransfer to its cognate RR BAS0540. In addition to the kinase, BAS0540 could also accept phosphate from physiologically important small molecule phosphodonors like acetyl phosphate (AcP) and carbamoyl phosphate (CP). Phosphorylation led to a striking enhancement in the DNA binding capability of BAS0540. The genes that could be potentially regulated by BAS0540 were identified by an *in silico* DNA motif search in the intergenic regions of *B*. *anthracis*. The predicted regulon comprised of 23 genes with a diverse set of functions. BAS0540 binding to the upstream regions of 5 putative regulon genes was shown *in vitro*. Further, we overexpressed the RR BAS0540 in *B*. *anthracis* under an IPTG inducible Pspac promoter, which led to a significant increase in the cell length, thereby imparting a filamentous phenotype to the bacteria. Moreover, it also caused a discernible decrease in the sporulation efficiency of the bacteria. Thus, our study characterizes a novel TCS of *B*. *anthracis* and provides insights into its role in the physiology of the pathogen.

## Materials and Methods

### Materials

*Escherichia coli* strains DH5α and BL21 were used as cloning and expression hosts, respectively. *E*. *coli* strain GM2929 (dam^-^dcm^-^) was used to propagate and isolate DNA for *B*. *anthracis* electroporation. *E*. *coli* strains were grown in Luria-Bertaini (LB) medium, supplemented with antibiotics, ampicillin (100μg/ml) and kanamycin (50 μg/ml) wherever required. An avirulent *Bacillus anthracis* Sterne 34F2 strain (pXO1^+^ pXO2^-^) was used in this study and cultured in Brain heart infusion (BHI) or sporulation broth (SB) medium supplemented with chloramphenicol (5μg/ml) wherever required. The growth of BAS0540 overexpressed *B*. *anthracis* strain was always supplemented with 1mM IPTG. While expression vector pET28a+ from Novagen (Madison, WI, USA) was used for heterologous gene expression, an *E*. *coli*-*Bacillus* shuttle vector pHCMC05 harboring an inducible Pspac promoter donated by the Bacillus Genetic Stock Center was used for homologous gene expression. All the restriction enzymes and DNA polymerases were from New England Biolabs Inc. (Ipswich, MA, USA). Ni^+2^-NTA agarose resin was from Qiagen (Hilden, Germany). Nitrocellulose membrane was procured from MDI membrane technologies (Ambala Cantt., India). Isopropyl β-D-thiogalactopyranoside (IPTG), bovine serum albumin (BSA) and antibiotics were from USB chemicals (Affymetrix, Ohio, USA). Anti-histidine mouse monoclonal, alkaline phosphatase-conjugated IgG, HRP-conjugated IgG, FITC-labeled IgG were from Sigma-Aldrich (St. Louis, MO, USA). Bradford reagent for protein estimation was from Bio-Rad (CA, USA). The oligonucleotides used in this study were synthesized from Sigma-Aldrich (St. Louis, MO, USA). [ɣ-^32^P]-ATP was obtained from BRIT (Hyderabad, India).

### Bioinformatics analysis

BAS0540 and BAS0541 were identified in the *B*. *anthracis* genome from KEGG [16], NCBI and P2CS [17] databases. While the operonic organization of BAS0540 and BAS0541 was checked by DOOR database [18], domain architecture of the proteins was determined using CDD (NCBI) [19] and SMART database [20]. BLAST analysis was performed on BAS0540 and BAS0541 to determine homologs in other pathogenic Firmicutes.

### Reverse transcription PCR

*B*. *anthracis* was grown up to exponential phase (O.D._600nm_ ~ 0.6) and total RNA was isolated using TRI reagent (Sigma-Aldrich). After DNase treatment, cDNA was synthesized from the total RNA by High Capacity cDNA Reverse Transcription kit (Life Technologies). The total cDNA pool was used as the template (25 ng) with 15 pmoles of indicated primers **(Table 1)** in a 25 μl reaction mix to detect the operon organization. The reaction products were analyzed by 1% agarose gel electrophoresis followed by eithidium bromide staining.

**Table 1:** Primers used in the study.

S.No	Study/Gene	Forward Primer 5’-3’	Reverse Primer 5’-3’
1	Cloning/BAS0540	GGCCCCATGGGCATGCGCTTACTCGTAG	GGCCCTCGAGTTGTTCTTTTAATATATATCC
2	Cloning/BAS0541	GGCCCCATGGGCATGAAAAAGGGAAGTATG	GGCCCTCGAGTATTCTTTGATTTTTAGGAAG
3	Two-step RT-PCR/BAS0540-BAS0541	ATGCGCTTACTCGTAGTAGAAG	CAATATAGTCAATCGAATACGCGTC
4	qRT-PCR/ BAS0540	CGCGTGAAAGGGTTGGATT	GTTGTTAAACTACCACTTCGCCTTAA
5	EMSA/BAS0540	GAAATAACCGTATTATAGGAATTG	ATATTCTCACCTTCCACCATATTG
6	EMSA/BAS3758	GTACATCTTAGTGTAGACTTATACTTGGC	TCTTTGGCACCTCCTTCTTTATTTATACC
7	EMSA/BAS2952	AAAAAAATTCTTATAAATATAGAGG	TGTATTTTTCCTCCTTTAG
8	EMSA/BAS1041	AAAAAATCCCTCCTAATTTC	CTCCTCCCCTATGTTACTTTTATC
9	EMSA/BAS5106	GCTTTTCTTATATTACTATAATCTTTC	ATTCTCGCTCCTTTTATAACTTAATAG
10	Overexpression/ BAS0540	GGCCGGATCCATGCGCTTACTCGTAGTAG	GGCCCCCGGGTTGTTCTTTTAATATATATCC

### Cloning of BAS0540, BAS0541FL, and BAS0541T in pET28a+ vector

**Fig 1:**
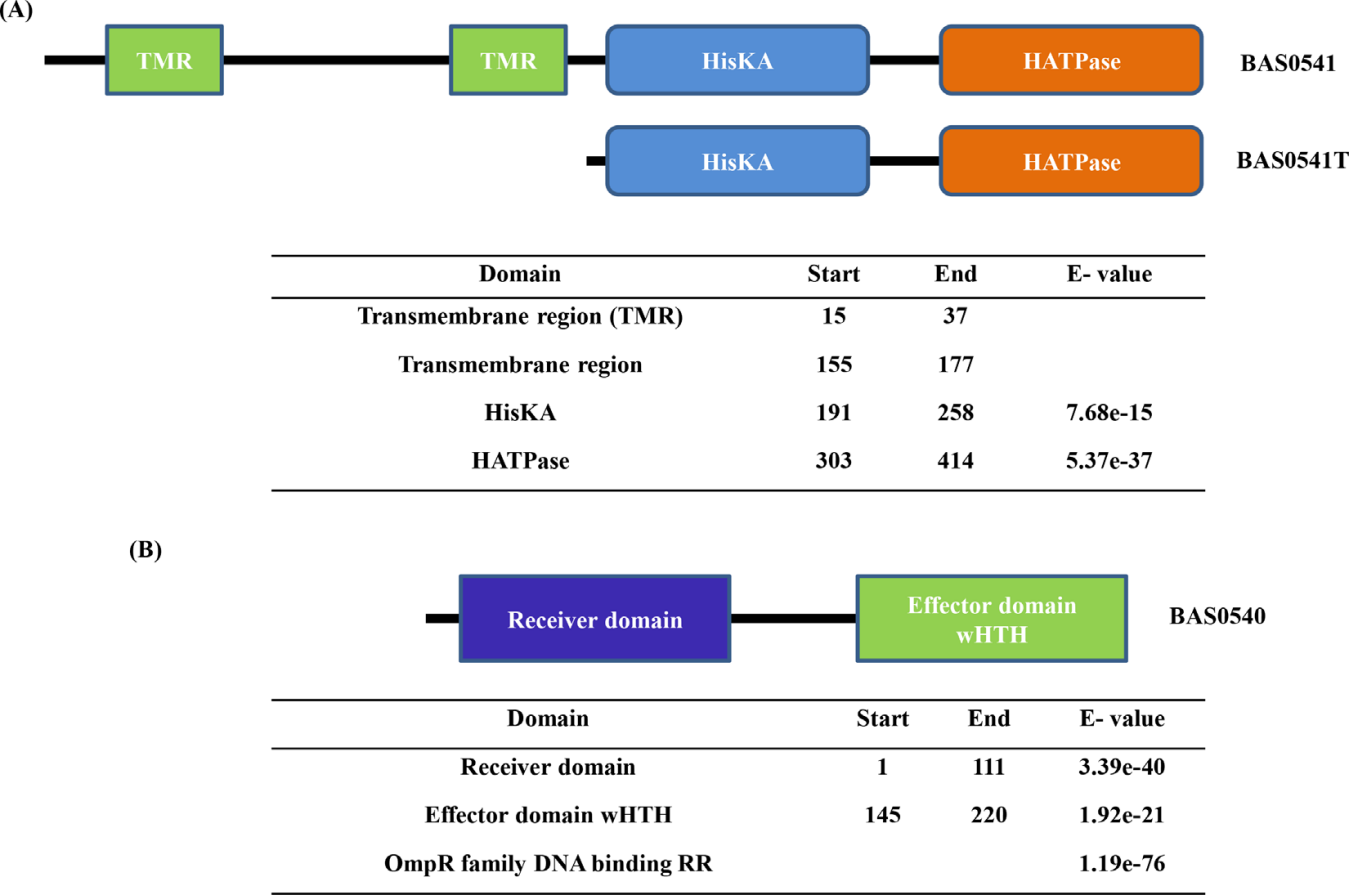
Bioinformatics analysis. (A, B) BAS0540 and BAS0541 follow the conventional modular paradigm and operonic organization of TCS proteins. The domain analysis for both the proteins was done using SMART database and CD search at NCBI. The E-values for the predicted domains are depicted in the tables. TMR-Transmembrane region.

While the RR was cloned as a full-length protein, the HK was cloned as both full-length (BAS0541FL) and truncated (BAS0541T) protein in which the N-terminal transmembrane domains were deleted. The region encompassing the two transmembrane domains (1–189 amino acid residues) was deleted, such that only the catalytic core comprising of HisKA and HATPase (190–416 amino acid residues) was cloned, expressed and purified. The deletion mutant BAS0541T is depicted in **Fig 1A**. The ORFs corresponding to BAS0540, BAS0541FL and BAS0541T were PCR amplified using specific primers **(Table 1)** from *B*. *anthracis* genomic DNA. After restriction digestion, the amplicons were ligated into the *Nco*I and *Xho*I sites of the expression vector pET28a+ to obtain pET28a-0540, pET28a-0541FL and pET28a-0541T constructs. The constructs were then transformed into DH5α cells and the sequence of each construct was confirmed by automated dideoxy DNA sequencing.

### Expression and purification of BAS0540 and BAS0541T

The construct pET28a-0540 was transformed into *E*. *coli* BL21 (λDE3) cells, grown till early log phase (O.D._600nm_ ~ 0.4), induced with 1mM IPTG at 37°C, and harvested 6h after induction. Protein purification was done from the soluble fraction using Ni^+2^–NTA affinity chromatography as described by Qiagen. A number of *E*. *coli* expression host strains (C43, pLysS, Rosetta) were tried for expression of the full-length kinase. However, no expression was achieved. The pET28a-BAS0541T construct was transformed into BL21 (λDE3) cells, which were then grown till log phase at 37°C (O.D._600nm_ ~ 0.6) and induced with 1 mM IPTG at 18°C for 16 h. The protein was purified from the soluble fraction by Ni^+2^-NTA affinity chromatography. The identity of all the recombinant proteins was confirmed by immunoblotting using anti-histidine antibody. Bradford reagent was used to determine the concentration of the proteins.

### Raising polyclonal sera against rBAS0540 and rBAS0541T in Swiss-albino mice

10 μg each of rBAS0540 and rBAS0541T were injected separately into Swiss-albino mice (3–4 weeks old) along with complete Freund’s adjuvant, which was followed by boosters with incomplete Freund’s adjuvant on 14^th^, 28^th^ and 42^nd^ day of immunization. The mice were bled after the 14^th^ day of each immunization and sera was collected. For both the proteins, endpoint titer was determined by Enzyme-linked immunosorbent assay (ELISA) which was expressed as the reciprocal of the endpoint dilution. All experiments involving mice were done in accordance with the guidelines of Jawaharlal Nehru University Institutional Animal Ethical Committee (JNU-IAEC). This study was conducted with the approval of JNU-IAEC.

### *In vivo* expression of BAS0540

#### Confocal microscopy

*B*. *anthracis* cells were grown up to exponential phase (O.D._600nm_ ~ 0.6) and harvested by centrifugation. All the washings were done with 1X PBS for 10 minutes at 4°C. The cells were washed twice, fixed with 4% paraformaldehyde followed by permeabilization using 0.5% Triton X-100 for 30 min at RT. After three washes, the cells were incubated with 2% BSA for 30 min, washed twice and then incubated with 1:50 dilution of primary antibody (polyclonal anti-BAS0540 sera) O/N at 4°C. After three washes the cells were incubated with 1:100 dilution of secondary antibody, FITC–labeled anti-mouse IgG for 2 h at RT, washed again and incubated with DAPI (5 μg/ml) to stain nuclear DNA for 5 min at RT. After a single wash, they were mounted on slides and visualized by Olympus FluoView FV1000 Laser Scanning Confocal Microscope. Successive z-stack sections were taken. Cells incubated with pre-immune sera, only secondary antibody, and no permeabilization were taken as negative controls.

#### qRT-PCR

qRT-PCR was done to assess the growth phase specific differences in BAS0540 transcript levels. Total RNA was isolated from *B*. *anthracis* Sterne strain grown in BHI medium at 37°C to an O.D._600nm_ of ~ 0.3, 0.6, 0.9 and 1.2 corresponding to the early exponential, exponential, late exponential and onset of stationary phase, respectively. Total RNA was isolated using TRI reagent, followed by RNase-free DNase treatment (Qiagen) and genomic DNA contamination check by PCR using gene specific primers. 1 μg of RNA was used to synthesize cDNA using the High Capacity Reverse transcription kit (Life technologies). A 10 μl reaction mix containing 5 μl 2X SYBR green master mix (Life technologies), 1 μl of 1/10 diluted cDNA (= 5 ng) and 40 nM gene specific primers (primers were designed using Primer Express software, version 2.0 (Applied Biosystems)) was used. The PCRs were run in ABI 7500 using a program described previously [21]. Thermal dissociation curves were analyzed post PCR amplification to detect non-specific amplification. cDNA dilution curves were generated and the individual real time PCR efficiencies were calculated by measuring the values of the slopes. Owing to its constitutive expression at the tested conditions, DNA gyrase was used as an endogenous control for data normalization. Normalized Ct values, mean with SEM are plotted against different growth stages.

#### Immunoblotting

*B*. *anthracis* cells were grown in BHI medium (O.D._600nm_ ~ 0.3, 0.6, 0.9 and 1.2) and were harvested and processed as described before [21]. Briefly, whole cells were pelleted, washed twice and then subjected to sonication for 20 min at 30% amplitude (2 mm microtip, 750W Sonic Vibra Cell Sonicator) which was followed by two centrifugation steps at a high speed of 13000 rpm for 30 min at 4°C. While the supernatant served as the soluble/cytoplasmic fraction, the pellet represented the cell wall plus the membrane fraction. The soluble fraction was subjected to immunoblotting using BAS0540 specific polyclonal antisera. Immunoblotting using antibodies against DNA gyrase of *B*. *anthracis* was used as a control for cytoplasmic protein in sub-cellular fractionation experiments. Equal protein loading was confirmed by quantifying the total protein content in each lysate by Bradford reagent and coomassie staining. 1:5,000 dilution of polyclonal anti-BAS0540 sera and 1:10,000 dilution of alkaline phosphatase conjugated IgG secondary antibody was used. The blot was developed by adding NBT/BCIP as the substrate solution.

### Autophosphorylation and phosphotransfer assay

Phosphorylation assays were performed as described previously [22]. Briefly, 3 μg of BAS0541T was incubated in phosphorylation buffer (100 mM Tris HCl [pH 8], 200 mM KCl, 4 mM MgCl_2_, 20 mM DTT, 0.1 mM EDTA, 3.5% glycerol, 2.5 μM ATP, 5 μCi of [ɣ-^32^P]ATP) in a final volume of 30 μl for 5, 15, 30 and 60 minutes at 37°C. The cold and radioactive ATP were mixed and the reaction was started by adding this ATP mixture and stopped by the addition of 5 μl of 6 X SDS-loading dye. While for cognate phosphotransfer, 3 μg of both the proteins were incubated, for non-cognate phosphotransfer, 5 μg of BAS0540 was mixed with either 3 μg of WalKc(PH) [21] or 3 μg of BAS5201 (annotated as DesK), in the phosphorylation buffer in a final reaction volume of 30 μl. Both the reactions were started by adding the radiolabelled ATP mixture. The reaction mixtures were analyzed by a 12% SDS-PAGE and the ^32^P labeled bands were visualized by autoradiography. For EMSA, BAS0540 and BAS0541T were incubated together in an appropriate ratio for 30 min at 37°C followed by addition of BAS3758 radiolabelled probe and incubation for 20 min at RT. The reaction mixtures were electrophoresed on 8% native polyacrylamide gels at 4°C followed by drying under vacuum at 70°C for 60–90 min and visualization and analysis of the radiolabelled probes and protein- DNA complexes on a Typhoon FLA 9500 phosphorimager. BAS0540 without pre-incubation with BAS0541T was used as a negative control for the phosphotransfer reaction.

### Autophosphorylation of BAS0540 by small molecule phosphoryl donors

BAS0540 (20 μg) was incubated with 60 mM carbamoyl phosphate (CP) and acetyl phosphate (AcP) (Sigma) in 40 μl of phosphorylation buffer (50 mM Tris HCl [pH 7.5], 50 mM KCl, 20 mM MgCl_2_, 20 mM DTT) for 15, 30, 45, 60 and 90 min at 37°C. 40 mM EDTA was used to stop the reaction since it arrests any further phosphorylation. The phosphorylation status was confirmed on 12% native PAGE followed by coomassie staining. Intensity analysis was done by ImageJ 1.45S software [23]. Only protein with no phosphodonor in the reaction mixture served as a negative control.

### Regulon Prediction

The study by de Been *et al* [15] suggested that BAS0540-BAS0541 could be similar to CiaRH of *Streptococcus pneumoniae*. This prompted us to determine the homology between DNA binding domains of CiaR and BAS0540 by BLAST. Swiss Model [24] was used to model the DNA binding domains of the two proteins and structural superposition was done using PyMOL. A high similarity between the two DNA binding domains led us to hypothesize that the two domains might bind to a similar DNA sequence. Thus, the consensus DNA binding site of CiaR- NTTAAGNNNNNTTTAAG of *Streptococcus pnuemoniae* [25] was used to perform an *in silico* motif search in the intergenic regions of complete *B*. *anthracis* genome using the DNA pattern search tool available at http://rsat.ulb.ac.be/genome-scale-dna-pattern_form.cgi [26] allowing 0 or 1 mismatch. The stringency of the search was maintained by allowing only one of the direct repeats to harbor a mismatch at one given time, keeping the other direct repeat exactly the same. A list of genes constituting the putative regulon of BAS0540 was obtained. Functional predictions for the regulon genes were made by analysis of the constitutive domains using Uniprot and NCBI CD search. Promoter predictions were done using PromBase [27] and Bprom program (Softberry) wherever needed. We also performed a search by allowing two mismatches at a time, one in each direct repeat; however, at no instance, did we allow the two Gs at positions 6 of the direct repeats to convert to any other nucleotides simultaneously (data not shown).

### Electrophoretic mobility shift assay (EMSA)

The probes for EMSA corresponding to the upstream regions of different regulon genes determined above were prepared by PCR using the designed primers **(Table 1)**. The amplified fragments were purified using gel extraction columns (MDI technologies) and labeled at their 5’ ends using T4 polynucleotide kinase and [ɣ-^32^P]ATP (3500Ci/mmol) at 37°C for 45–60 min, followed by inactivation of the enzyme at 70°C for 10 min. Unincorporated nucleotides were removed using nanosep centrifugal devices (Pall corporation). The binding reaction was initiated by adding 1–1.5 pmoles of each labeled probe to the phosphorylated or non-phosphorylated protein in a reaction volume of 40 μl binding buffer (10 mM Tris HCl [pH 7.5], 50 mM NaCl, 10 mM MgCl_2_, 20 mM DTT, 10% glycerol, 0.5 μg poly (dI-dC)) and incubated at 25°C for 25 min. Varying amounts of BAS0540 were phosphorylated by 60 mM carbamoyl phosphate in the binding buffer for 45 minutes at 37°C prior to the initiation of the binding reaction. In order to test the specificity of the BAS0540-DNA interaction, specific and non-specific competition assays were performed. Briefly, for all competition experiments 25- to 200-fold molar excess of unlabeled specific or non-specific DNA (25X, 50X, 100X and 200X) was added to the binding buffer containing the phosphorylated BAS0540 prior to the addition of radiolabelled probe. The NBS DNA lacking the defined consensus binding site of BAS0540 RR served as a negative control. NBS is basically a 250bp DNA probe from the upstream region of yet another two component system BAS2056-BAS2057 of *B*. *anthracis*. In order to demonstrate the effect of changes in the DNA binding sequence on the DNA binding of BAS0540, if any, the probes with two mismatches, one in each direct repeat were also tested. The reaction mixtures were electrophoresed on 8% native polyacrylamide gels (29:1 acrylamide: bisacrylamide ratio) in 0.5 X TBE buffer at 4°C followed by drying under vacuum at 70°C for 60–90 min and visualization and analysis of the radiolabelled probes and protein- DNA complexes on a Typhoon FLA 9500 phosphorimager.

### Electroporation of *B*. *anthracis* competent cells with Pspac-BAS0540 construct

Electrocompetent *B*. *anthracis* cells were made using the protocol described by Koehler *et al* [28]. To a 200 μl aliquot of electrocompetent *B*. *anthracis* cells, 10 μg DNA was added in a pre-chilled cuvette of 0.2 cm electrode gap (Bio-Rad). Electroporation was carried out at 2.0 kV, 200Ω and 25 μF using a Bio-Rad Gene Pulser with a pulse controller and capacitance extender. After providing the pulse, the cells were incubated on ice for 5 min, followed by diluting them with 1 ml of BHI medium supplemented with 10% glycerol, 0.4% glucose, and 10mM MgCl_2_. The mixture was then incubated at 37°C for 2 h, followed by plating it on BHI agar plate containing 5 μg/ml chloramphenicol. The plates were incubated for 20 h at 37°C.

### BAS0540 overexpression in *B*. *anthracis*

The ORF corresponding to BAS0540 was PCR amplified from the genomic DNA of *B*. *anthracis* using specific primers **(Table 1)**. After double digestion, the amplicon was ligated to the *BamH*I and *Sma*I sites of pHCMC05 vector, thereby placing BAS0540 ORF under an IPTG inducible Pspac promoter. The Pspac-BAS0540 construct was then electroporated into *B*. *anthracis* as described above. The transformants were screened by colony PCR using *cat* cassette amplification. The positive transformants were cultured and sub-cultured in BHI medium supplemented with 5 μg/ml chloramphenicol and 1mM IPTG. *B*. *anthracis* cells harboring only pHCMC05 vector served as negative control.

### DIC microscopy

Both wild type *B*. *anthracis* and BAS0540 overexpressed strain were grown up to OD_600nm_~ 0.6 in BHI broths and subsequently 10 μl of each culture was mounted on glass slides for DIC microscopy. For BAS0540 overexpressed strain, the broth was supplemented with 5 μμg/ml chloramphenicol and 1mM IPTG. The mean and standard deviation of the mean of 10 cells from three independent experiments are plotted.

### Spore formation and titer determination

100 ml sporulation broths were inoculated with 1% inoculum of overnight grown primary cultures of wild type and BAS0540 overexpressed strain of *B*. *anthracis*, each, followed by incubation at 37°C for 24 h with shaking at 140 rpm. At the end of 24 h, the cultures were diluted to 1/100 and 10ul was plated on BHI agar plate to determine the initial CFU/ml. The cultures were then subjected to heat treatment at 65°C for 2 h to ensure that all the vegetative cells are killed and only spores remain. The spores were then washed 5 times with 1X PBS and finally each spore pellet was dissolved in 500ul of 1X PBS. 10 μl of 1/100 diluted spores was then plated on BHI agar plate, followed by incubating the plates at 37°C for 16–18 h. For BAS0540 overexpressed *B*. *anthracis* strain, BHI agar plate was supplemented with 5 μg/ ml chloramphenicol and 1mM IPTG. The normalized spore titer was calculated for both wild type and BAS0540 overexpressed strain. Mean with SD are plotted.

## Results

### BAS0540 and BAS0541 follow the conventional modular paradigmof TCS proteins and are transcriptionally coupled

Using the NCBI genome and protein databases, BAS0540 and BAS0541 encoding a RR and HK, respectively, were identified on the chromosome of *B*. *anthracis* Sterne strain. Domain architecture of the two proteins revealed that the RR BAS0540 mirrors a traditional RR, having an N-terminal receiver domain-REC that belongs to the REC superfamily and a C-terminal DNA binding domain-trans_reg_C that belongs to the winged Helix Turn Helix superfamily, typical of the OmpR family of RRs. The HK BAS0541 consists of two transmembrane domains at the N-terminal, and a HisKA (DHp) and HATPase (CA) at the C-terminal. The two transmembrane domains were predicted using TMHMM. The E-values for these domain predictions are depicted in **Fig 1A and 1B**. A study by de Been *et al*. suggested that BAS0540-BAS0541 TCS is similar to the CiaRH of *Streptococci* [15]. We made an analogous observation where we found that the closest ortholog of CiaRH in *B*. *anthracis* was BAS0540-BAS0541. Operonic organization of BAS0540 and BAS0541 as predicted by DOOR database was confirmed by reverse transcriptase PCR. The two genes BAS0540 and BAS0541 are adjacent to each other in the same orientation on *B*. *anthracis* genome and are co-transcribed. While the forward primer binds at the very start of BAS0540 ORF, the reverse primer was designed such that it spans 50 bp of the downstream gene BAS0541, thus making the PCR amplicon as 723 nucleotides in size **(S1 Fig).**

### Cloning, expression and purification of BAS0540 and BAS0541T

**Fig 2:**
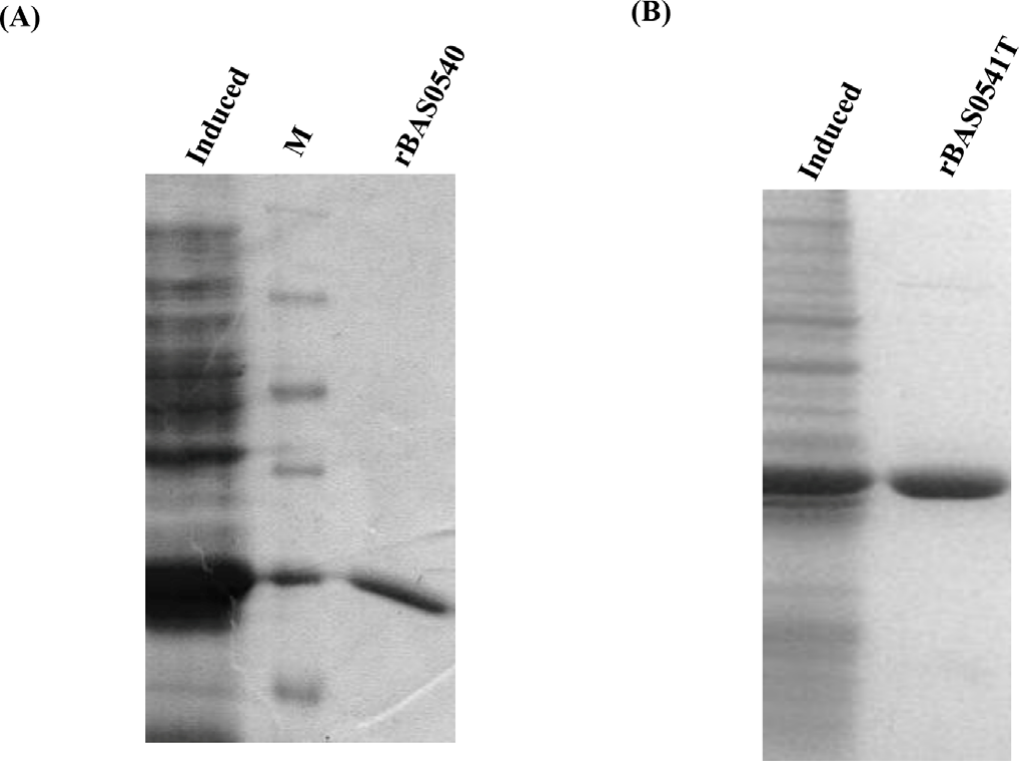
Expression and purification of BAS0540 and BAS0541T. (A, B) Purified BAS0540 and BAS0541T. Both proteins were purified from the soluble fraction by Ni^+2^ –NTA affinity chromatography.

All the ORFs i.e. BAS0540, BAS0541FL, and BAS0541T were cloned in the expression vector pET28a+ which adds a 6X-his tag to their C-terminus. Both BAS0540 and in-frame partial deletion of BAS0541FL, BAS0541T were readily expressed and purified from the soluble fraction of BL21 (DE3) cells. The proteins were analyzed on 12% SDS- PAGE and migrated at their expected molecular weights, i.e. 25 kDa and 26 kDa, respectively **(Fig 2A and 2B)**. Expression of membrane bound sensor kinases as full-length proteins, in most of the cases, has been a difficult task. Exploiting the fact that the transmembrane domains of the sensor kinase can be removed without abrogating its functionality [29–31], we truncated the kinase, uncoupling its sensor and catalytic domains.

### *In vivo* expression and growth phase independent abundance of BAS0540

#### Confocal Microscopy

**Fig 3:**
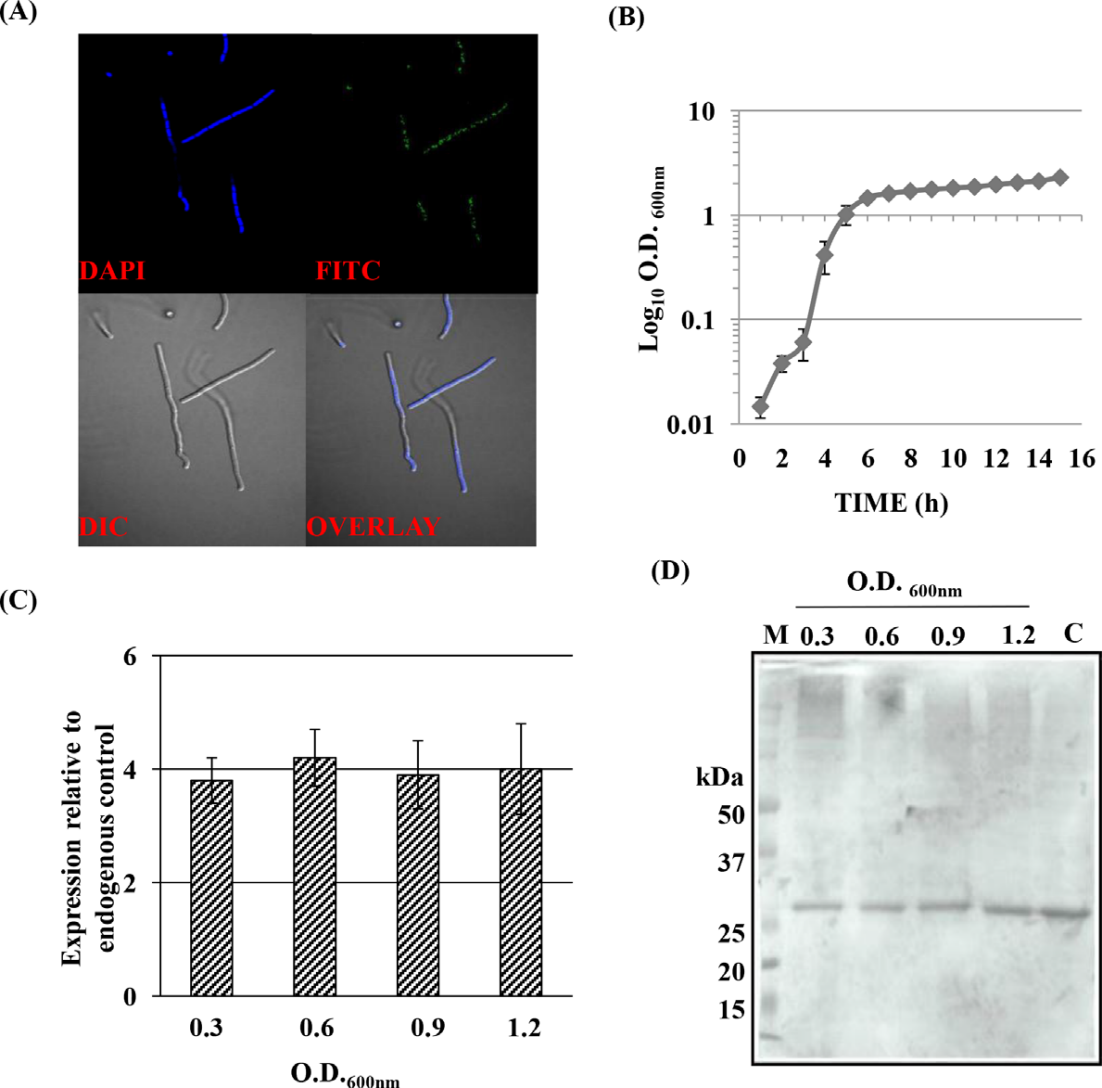
*In vivo* expression and growth phase independent abundance of BAS0540 in *B*. *anthracis*. (A) Confocal microscopy. Cells treated with 1:50 dilution of anti-BAS0540 polyclonal sera followed by treatment with 1:100 dilution of FITC–labeled anti-mouse IgG secondary antibody. (B) Growth curve of *B*. *anthracis* in BHI broth. (C) qRT-PCR for BAS0540 from total RNA of *B*. *anthracis* (O.D._600nm_~ 0.3, 0.6, 0.9, 1.2). Normalized Ct values are plotted against different growth stages. Mean with SEM values, from two independent experiments carried out in triplicates are shown. (D) Immunoblotting for growth stage specific endogenous expression of BAS0540 using anti-BAS0540 antisera. + denotes recombinant BAS0540. M- Molecular weight standards in kDa.

The *in vivo* expression of BAS0540 in *B*. *anthracis* was confirmed by confocal immunofluorescence microscopy using anti-BAS0540 polyclonal antisera **(Fig 3A)**. Cells treated with pre-immune sera and only secondary antibody did not show any fluorescence **(S2 Fig)**. Z-stack analysis excluded any possibility of fluorescence due to non-specific adherence of the FITC labeled secondary antibody on the surface of cells **(S2 Fig)**.

#### Quantitative real time PCR

The growth curve of *B*. *anthracis* in BHI medium was monitored **(Fig 3B)** and four different growth points were selected as early exponential (O.D._600nm_ ~ 0.3), mid exponential (O.D._600nm_ ~ 0.6), late exponential (O.D._600nm_ ~ 0.9) and onset of stationary phase (O.D._600nm_ ~ 1.2), respectively. BAS0540 mRNA could be detected throughout the growth period, indicating that BAS0540 was transcribed throughout the growth phases **(Fig 3C)**.

#### Immunoblotting

Growth phase dependent changes, if any, in the endogenous expression of BAS0540 were ascertained by immunoblotting of the cytoplasmic/soluble fractions of *B*. *anthracis* lysates. Endogenous BAS0540 expression could be detected at all the phases of the growth, displaying a perfect corroboration with qRT-PCR data. The endogenous BAS0540 migrated at the same size as did the recombinant BAS0540 i.e. at ~ 25 kDa since the difference in the molecular weights of endogenous and recombinant BAS0540 was just ~ 1.1 kDa **(Fig 3D)**.

However, we could not detect HK BAS0541 in the cell lysate of *B*. *anthracis*. Though RR and HK are co-transcribed, it has been frequently observed that at the protein level, RRs are more abundant when compared to their cognate HKs. Li *et al* used ribosome profiling to explain that this difference could be attributed to the differential expression of the components of functional modules like TCSs and toxin-antitoxin systems which is in accordance with their hierarchal role. This expression pattern is applicable to each of the 26 TCSs of *E*. *coli* and can be extrapolated to other TCSs as well, thus making RRs in excess to their cognate HKs in the cell [32].

### Autophosphorylation of BAS0541 and phosphotransfer to BAS0540

**Fig 4:**
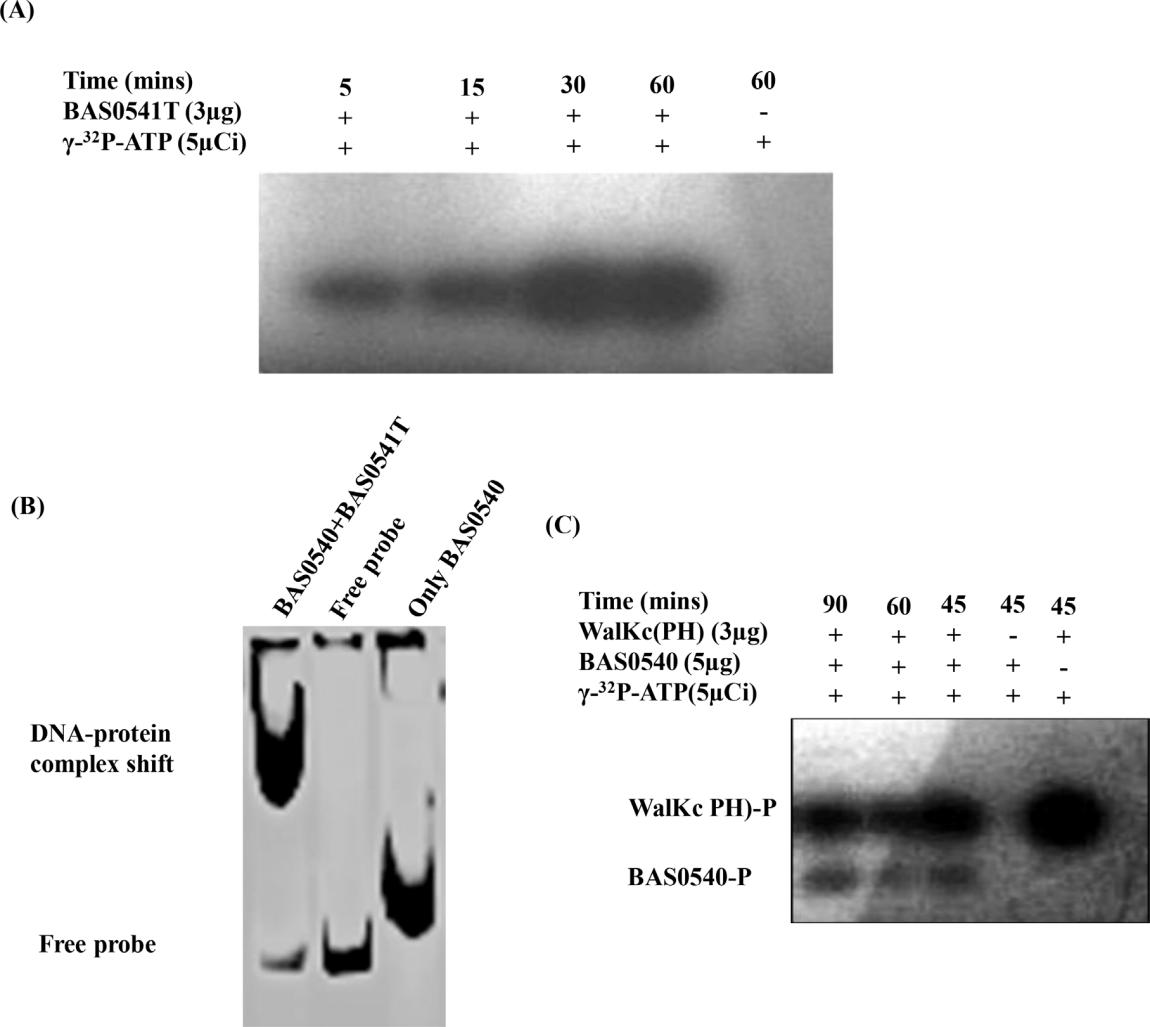
Autophosphorylation of BAS0541T, cognate (BAS0540-BAS0541) and non-cognate phosphotransfer (WalKc(PH)-BAS0540). (A) Autophosphorylation of BAS0541T was observed within 5 min of [ɣ-^32^ P]ATP addition and increased with time. (B) EMSA employing BAS0540 pre-incubated with BAS0541T prior to radiolabelled BAS3758 probe addition. A more pronounced shift compared to BAS0540 not pre-incubated with BAS0541T indicates the presence of phosphorylated BAS0540 species. (C) Phosphorylated RR was detected only after 45 min of [ɣ-^32^ P]ATP addition in non-cognate phosphotransfer from WalKc(PH) to BAS0540. The level of phosphorylation was very low. The reaction mixtures were analyzed by a 12% SDS–PAGE, followed by autoradiography. Only response regulator and only kinase controls were put wherever needed.

Autophosphorylation at a conserved histidine residue in HK and subsequent transfer to a conserved aspartate residue in RR is the basis of TCS signaling mechanism. The biochemical assays depicting autophosphorylation and phosphotransfer were carried out using the truncated kinase BAS0541T, also connoting that the N-terminal transmembrane domains are not imperative for it to function as a kinase. Autophosphorylation was assayed by incubating the purified BAS0541T with radiolabelled ATP mixture and could be detected as early as 5 min post ATP addition which subsequently increased reaching a maximum at 60 min **(Fig 4A)**. Phosphotransfer reaction was assessed by incubating BAS0540 and BAS0541T together with radiolabelled ATP mixture. It should be noted that while the full-length RR BAS0540 is a 25 kDa protein, the truncated HK used in the phosphotransfer assay BAS0541T is 26kDa. A mere difference of 1 kDa between the two proteins hampered their resolution as two separate bands on 12% SDS-PAGE and thus we could not observe two distinct radiolabelled bands by autoradiography **(S3 Fig)**. Such phosphotransfer reactions involving HK and the cognate RR of approximately same molecular weights have been attempted previously as well [30]. However, at the end of 60 min, the amount of phosphorylated HK (40%) in the autophosphorylation reaction was more than the phosphorylated RR (30%) in the phosphotransfer reaction **(S3 Fig)**. The DNA binding ability of BAS0540 enhances substantially upon phosphorylation, an attribute that we exploited for providing a strong evidence for successful phosphotransfer reaction between BAS0541T and BAS0540. A more pronounced shift was observed when BAS0540 was pre-incubated with BAS0541T, prior to radiolabelled probe addition **(Fig 4B)** as compared to the unphosphorylated BAS0540 (BAS0540 not pre-incubated with BAS0541T), thus corroborating the presence of phosphorylated BAS0540 species upon incubation with the cognate kinase.

Non-cognate phosphotransfer reactions behaved considerably differently from cognate phosphotransfer reactions. These reactions were monitored for 90 min. When BAS0540 was incubated with an HK of the same family, WalKc(PH) [21] the RR was phosphorylated only after 45 minutes post ATP addition. The phosphotransfer increased up to 90 min; however the level of phosphorylation was very low **(Fig 4C)**. In non-cognate phosphotransfer reaction from an HK of a different (NarL) family, we could not observe any phosphorylation of the RR even at the end of 90 min. Since the time taken for this phosphotransfer was much more as compared to the transfer between BAS0541 and BAS0540, it can be categorized as a non-specific reaction and therefore most likely of no physiological relevance.

### Phosphorylation of BAS0540 by small molecule phosphoryl donors

**Fig 5:**
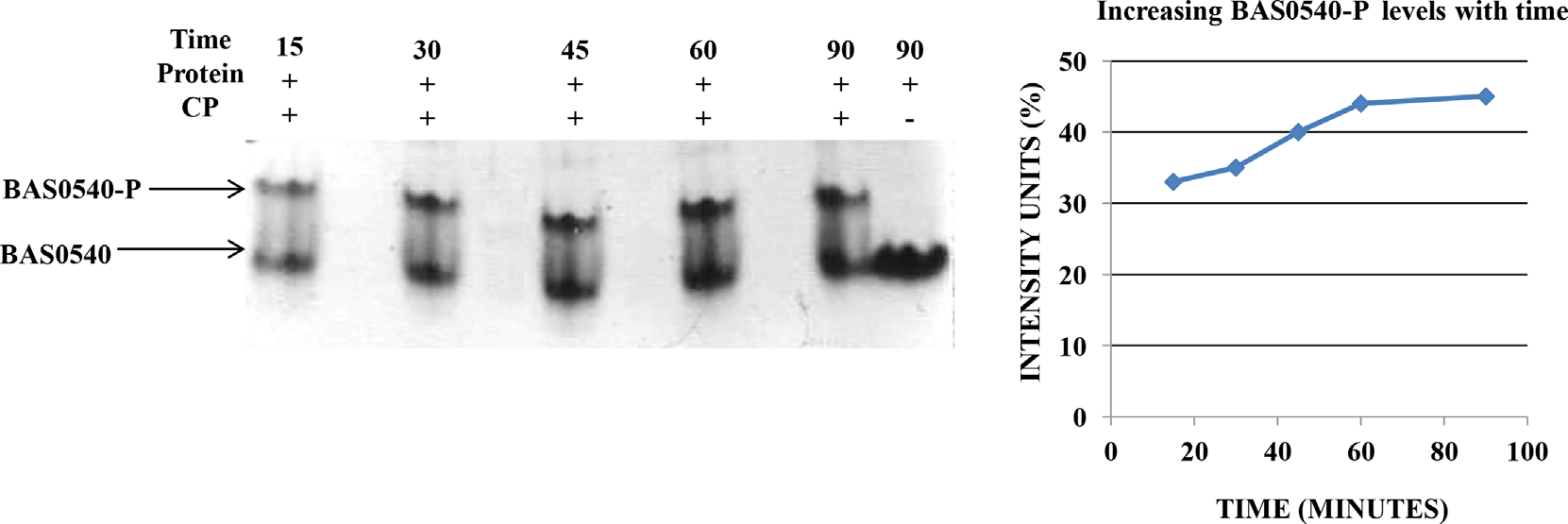
Phosphorylation of BAS0540 by Carbamoyl phosphate (CP). (A) BAS0540 could be phosphorylated with CP which increased with time, reaching a maximum of 40–45%. Intensity analysis was done using ImageJ 1.45S software. Intensity units (% RR) are plotted against time in minutes. 12% native PAGE followed by coomassie staining and intensity analysis was used to analyze the phosphorylation status of BAS0540.

BAS0540 was found to catalyze its own phosphorylation *in vitro* using physiologically relevant small molecule phosphodonors, AcP and CP **(S4 Fig; Fig 5A)**. The autophosphorylation of BAS0540 reached a maximum at 45 minutes, after which the reaction plateaued. The maximum phosphorylated BAS0540 was estimated to be 40–45% from two independent experiments **(Fig 5A)**. The phosphorylation status of the RR was confirmed by an additional band in the native PAGE with a slower mobility indicating the dimeric form of the RR (when phosphorylated, most of the RRs undergo dimerization), which was clearly absent in the only RR control lacking any small molecule phosphodonors [33–35]. This phosphorylation of BAS0540 was acid stable and base labile [data not shown] and required Mg^+2^ ions indicating that the residue phosphorylated was an aspartate. Asp50 was identified as the most probable phosphoacceptor residue by aligning BAS0540 with its homologs from other Firmicutes **(S4 Fig)**.

### Regulon prediction

CiaRH from *S*. *pneumoniae* was speculated to be the homolog of BAS0540-0541 in *B*. *anthracis* [15]. A significant similarity of 79% was observed between the DNA binding domains of CiaR and BAS0540. DNA binding domains of the two proteins were modeled which gave QMEAN values of -2.71 and -1.6 respectively, followed by structural alignment with an excellent RMS deviation of 0.049 (data not shown). The above results prompted us to hypothesize that the two proteins might bind to similar DNA sequences. Using the methodology described (0 or 1 mismatch) (2.10), we obtained a list of 30 genes out of which 23 could be assigned functional annotations **(Table A in S1 Text)**. We functionally classified the candidates into 8 categories: 5 of them were transcriptional regulators, 2 were involved in cell wall metabolism, 9 genes encoded for transporters, 4 of them were involved in different stress responses, 7 were involved in general metabolism and one gene each in cell division, self immunity and antimicrobial resistance **(S5 Fig)**. The functional classification of the putative candidates of BAS0540 regulon indicate that it might regulate a diverse set of functions; however a definitive role can be assigned to this system with high confidence only after a detailed transcriptomics study involving overexpression of the RR BAS0540, currently being conducted by our group. Absence of the classical virulence factors in the putative regulon is notably contrasting to the regulon of CiaRH in *Streptococcus*, which includes HtrA, the major virulence factor of the pathogen [36]. The list obtained by allowing 2 mismatches, one in each direct repeat consisted of more number of genes (data not shown). For comparing the affinity of BAS0540 for probes with 0/1 and 2 mismatches, 2 genes from this list were selected for binding studies. As the number of mismatches increased, the DNA binding ability of BAS0540 was found to decrease.

### DNA binding ability of BAS0540

**Fig 6:**
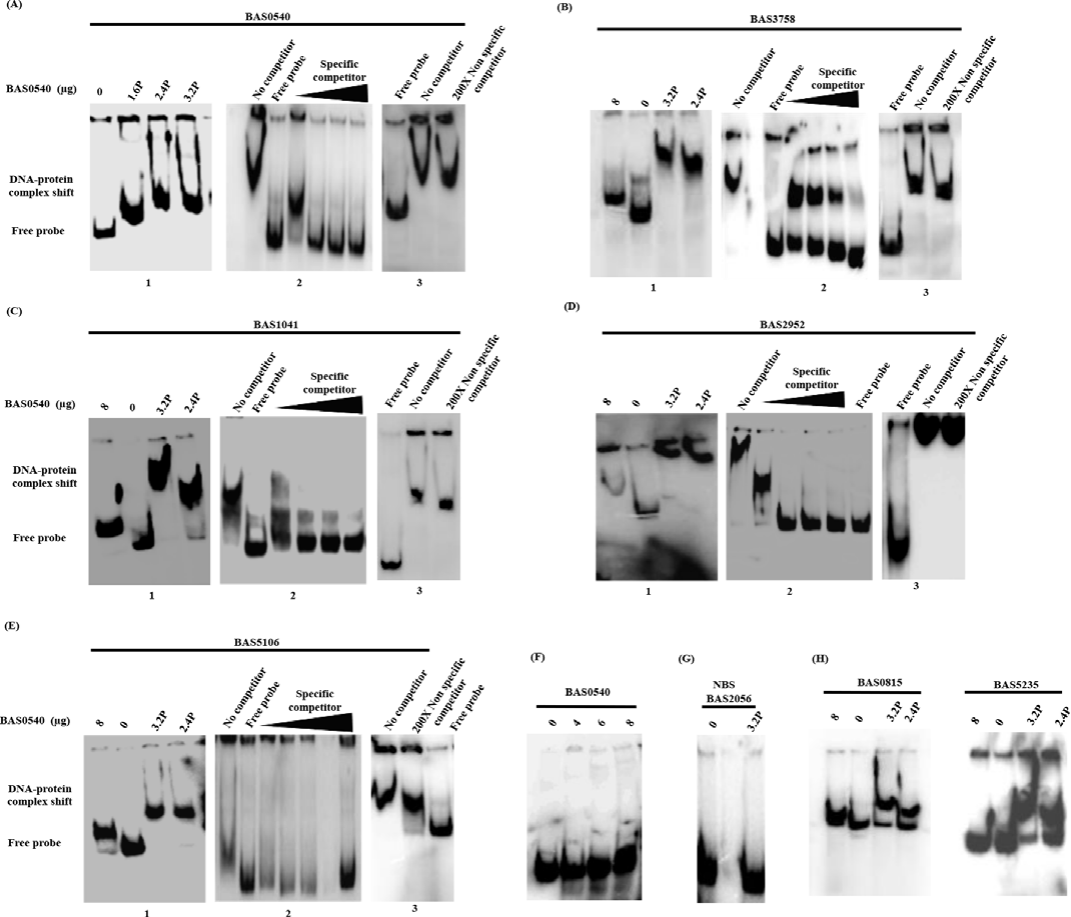
DNA binding ability of BAS0540. EMSA was used to check for BAS0540 binding to DNA by incubating increasing amounts of purified phosphorylated and unphosphorylated BAS0540 with upstream regions of 5 different candidate genes of the putative regulon containing the defined binding site. (A1, B1, C1, D1, E1) Phosphorylated and unphosphorylated RR BAS0540 incubated with radiolabelled probe of BAS0540, BAS3758, BAS1041, BAS2952 and BAS5106. (A2, B2, C2, D2, E2) Competition with molar excess (25X, 50X, 100X, and 200X) of unlabeled specific probe. (A3, B3, C3, D3, E3) Competition with 200X molar excess of unlabeled non-specific probe. (F) Increasing amount of unphosphorylated BAS0540 (4, 6 and 8 μg) incubated with BAS0540 probe. Such a gradient was put for other probes as well (data not shown). (G) BAS0540 incubated with NBS, a 250bp DNA probe that lacks the BAS0540 binding site. (H) BAS0540 incubated with probes (BAS0841 and BAS5235) containing 2 mismatches, one in each direct repeat. P indicates phosphorylated BAS0540 and was calculated by assuming ~ 40% phosphorylation by CP (40% of 4, 6 and 8 μg). Binding reactions were performed at RT and the reaction products were run on 8% polyacrylamide native gels in 0.5X TBE at 4°C. The gels were dried under vacuum at 70°C for 60–90 min. The radiolabelled probes and protein- DNA complexes were visualized with a storage phosphor screen and analyzed on a Typhoon FLA 9500 phosphorimager. Phosphorylated BAS0540 could bind to all the five test probes. Competition experiments and no binding with NBS demonstrate the specificity of BAS0540-DNA interactions.

BAS0540 harbors a C-terminal DNA binding domain with a winged helix turn helix motif and thus, can be categorized under the OmpR family of RRs **(Fig 1B)**. To test its DNA binding activity, varying amounts of unphosphorylated and phosphorylated BAS0540 were incubated with DNA probes from upstream regions of five different genes [0/1 mismatch] containing its defined binding site **(Table 2)**. All the binding reactions were carried out in the presence of an excess of non-specific competitor DNA poly (dI-dC). With most of the probes, the unphosphorylated BAS0540 was able to cause only a slight shift and that too at the highest concentration tested, i.e. 8 μg, if at all **(Fig 6A1, 6B1, 6C1, 6D1, 6E1 and 6F1)**. Therefore, BAS0540 was phosphorylated for binding assays using small molecule phosphodonors. In most of the cases, 40% average phosphorylation of BAS0540 by CP, led to a discernibly strong complete shift due to formation of higher molecular weight protein-DNA complexes even at lower concentrations of total protein like 4 μg (data not shown) and 6 μg **(Fig 6A1, 6B1, 6C1, 6D1 and 6E1)**. Hence, phosphorylation of BAS0540 definitely enhanced its DNA binding activity. Considering an average of 40% phosphorylation by CP, the concentration of phosphorylated BAS0540 can be estimated to be approximately 1.6 μg, 2.4 μg and 3.2 μg **(Fig 6A1, 6B1, 6C1, 6D1 and 6E1)** when the total protein concentration was 4 μg, 6 μg, and 8 μg, respectively. In the competition experiments adding excess of unlabeled specific competitor DNA did progressively abolish the shift **(Fig 6A2, 6B2, 6C2, 6D2 and 6E2)**, whereas addition of non-specific unlabeled competitor probe did not affect the shift **(Fig 6A3, 6B3, 6C3, 6D3 and 6E3)**. BAS0540 did not exhibit any binding to the NBS probe **(Fig 6G)**, a 250 bp DNA fragment from the upstream region of another novel TCS of *B*. *anthracis* BAS2056-BAS2057. We are in the process of functionally characterizing this system as well. The RR of this TCS, BAS2056 exhibits binding to this 250bp DNA fragment, NBS, as seen by electrophoretic mobility shift assays (unpublished results). Thus, BAS2056 binds to its own upstream region and might regulate its own operon. Hence, we demonstrate here that BAS0540 did not bind to the promoter of a gene controlled by another TCS BAS2056-BAS2057 of *B*. *anthracis*. An incomplete shift was obtained with probes containing 2 mismatches **(Table 2)**, one in each direct repeat indicating that the affinity of BAS0540 was less for these probes **(Fig 6H)**.

**Table 2:** BAS0540 binding site in the upstream regions of putative regulon candidates^C^.

Gene	Sequence	Predicted function
BAS0540^a^	TTTAAGNNNNNTTTAAG	OmpR family response regulator
BAS1041^a^	TTTAAGNNNNNTTTAAG	CAAX protease self-immunity
BAS2952^a^	ATTAAGNNNNNTTTAAG	Heat induced stress protein, YflT
BAS3758^a^	TTTAACNNNNNTTTAAG	Cell division protein, FtsA
BAS5106^a^	TTTAAGNNNNNTTTAAG	RND family efflux transporter MFP subunit
BAS0815^b^	TTTGAGNNNNNTGTAAG	Small, acid-soluble spore protein
BAS5235^b^	CTTGAGNNNNNTTTAAA	Hypothetical protein, prespore-specific regulator
**Consensus**^d^	**NTTAAGNNNNNTTTAAG**	

a- Candidates of putative regulon with 0/1 mismatch.

b- Candidates with 2 mismatch at one time, one in each direct repeat.

c- All the binding sites were present in the intergenic region of *B*. *anthracis* chromosome.

d- Consensus drawn from the candidates with 0/1 mismatch^a^.

Direct repeats are underlined.

### Overexpression of BAS0540 manifests a filamentous phenotype to *B*. *anthracis*

**Fig 7:**
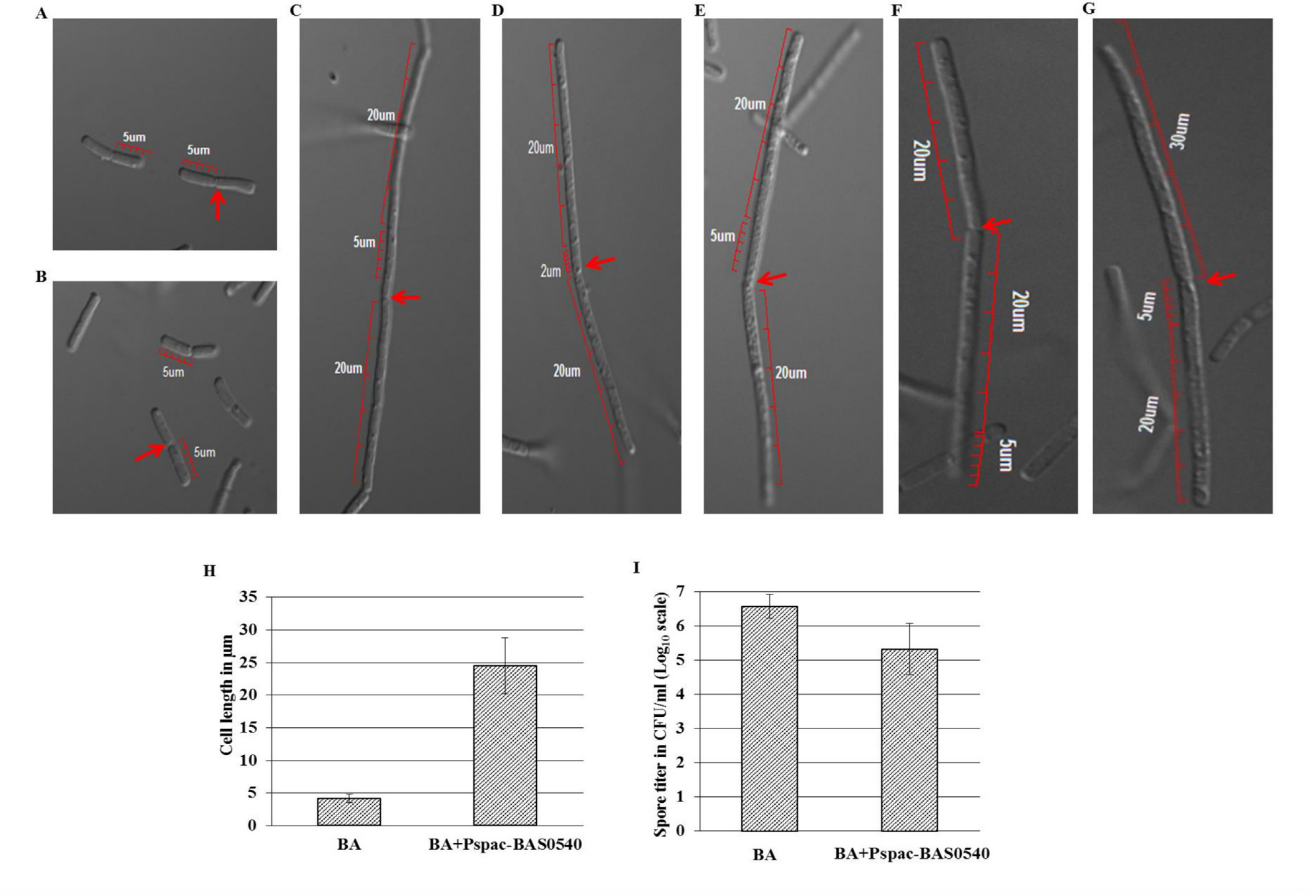
BAS0540 overexpression in *B*. *anthracis* and its effect on cell division and sporulation. DIC microscopy and sporulation titer determination (A) Wild type *B*. *anthracis*. (B) *B*. *anthracis* cells electroporated with pHCMC05 vector only. (C-G) BAS0540 overexpressed *B*. *anthracis* strain. (H) A 6-fold increase in the mean cell length of BAS0540 overexpressed strain was observed as compared to the wild type *B*. *anthracis*. Mean with SD are plotted. (I) A 16-fold reduction in the sporulation titer of BAS0540 overexpressed strain was seen as compared to the wild type *B*. *anthracis*. Mean with SD are plotted. Red arrows denote the septum between the two cells. An additional 2μm or 5μm scale bar has been added wherever required, to depict the cell length between 20 μm and 30 μm.

BAS0540 ORF was placed under an IPTG inducible Pspac promoter and introduced into *B*. *anthracis* using a shuttle vector pHCMC05, so as to determine its effect on the physiology of *B*. *anthracis*. However, we could observe a weak overexpression of only 1.9-fold, as checked by RT-PCR (data not shown). Differential Interference Contrast (DIC) microscopy revealed a striking difference in the morphology of *B*. *anthracis* cells overexpressing BAS0540, wherein overexpression of BAS0540 led to the elongation of cells, increasing the mean cell length by ~ 6 folds as compared to the wild type *B*. *anthracis* **(Fig 7H)**, thereby imparting them a filamentous phenotype **(Fig 7C–7G)**. To eliminate the possibility of this observation being an artifact of electroporation, cells electroporated with only vector DNA were also observed by DIC microscopy, which confirmed that this morphological change was indeed due to BAS0540 overexpression **(Fig 7B)**.

### Overexpression of BAS0540 decreases sporulation in *B*. *anthracis*

Sporulation, a starvation response, is an important event in the life cycle of *B*. *anthracis* since it helps the bacteria to evade and survive through the adverse conditions. Here we checked the effect of BAS0540 overexpression in *B*. *anthracis* on its sporulation efficiency and titer. BAS0540 overexpression led to a ~16-fold reduction in the sporulation titer **(Fig 7I)**. Further, we did not observe any difference in the germination of spores of wild type *B*. *anthracis* or BAS0540 overexpressing strain.

## Discussion and Conclusion

In the present study, RR BAS0540 and HK BAS0541 of *B*. *anthracis* were shown to form a functional TCS, displaying the classical properties of autophosphorylation and phosphotransfer. In a study by de Been *et al* [15], this TCS was indicated to exhibit similarity with CiaRH of *Streptococcus pnuemoniae*. In addition to the kinase, BAS0540 could also accept phosphate from small molecule phosphodonors. BAS0540 exhibited a growth phase independent abundance in *B*. *anthracis*, as seen by qRT-PCR and immunoblotting. The putative regulon predicted by an *in silico* approach comprised of 30 genes, of which 23 were annotated for their function. The DNA binding ability of phosphorylated BAS0540 was significantly greater than the unphosphorylated form as ascertained by the *in vitro* binding assays involving both the forms of BAS0540 and upstream regions of 5 candidates of its putative regulon, *ftsA*, BAS0540, *yflT*, BAS1041, and BAS5106. Further, BAS0540 overexpression in *B*. *anthracis* conferred a filamentous phenotype to the bacteria and also decreased its sporulation efficiency considerably, validating regulation of *ftsA* and connoting the role of this TCS in cell division and sporulation.

The rate and extent of phosphorylation of a RR is strikingly different in cognate and non-cognate phosphotransfer reactions. Though, non-cognate phosphotransfer reactions possess attributes like lower affinity, slower kinetics and seldom occurrence, all of which are important in avoiding the unwanted crosstalk that can occur between temporally and spatially co-existing TCSs in the cell, which can otherwise be detrimental [37–39], some of them have been categorized chiefly non-specific due to their extremely slow nature and requirement for an abnormally high concentration of the non-partner RR. The non-cognate phosphotransfer reaction monitored by us between WalKc(PH) and BAS0540, too, falls in the above category and hence cannot be most likely foreseen under the physiological conditions. Such physiologically irrelevant non-cognate reactions have been reported previously as well [40].

Many RRs can be autophosphorylated by physiologically relevant small molecule phosphodonors like AcP and CP; however, exceptions to this canonical paradigm do exist, as exemplified by CheB of *E*.*coli* [41], WalR of *B*. *subtilis* [42] and *S*. *aureus*, PhoP of *B*. *subtilis* [43], MtrA of *Mycobacterium tuberculosis* [44] and DrrB of *Thermotoga maritima* [45]. All of them cannot utilize AcP or CP to autophosphorylate themselves. The RRs of the OmpR/PhoB family are broadly grouped into two classes-one that are readily autophosphorylated and the other that are poorly phosphorylatable by small molecule phosphodonors. Moreover, there are some regulators that harbor different affinities for different donors, for e.g. Rrp2, an RR from *Borrelia burgdorferi* can accept phosphate from AcP but not from CP [46], SsrB of *Salmonella* has a higher affinity for phosphoramidate when compared to AcP [47]. Also, the inherent efficiency for autophosphorylation of different RRs by a particular small molecule phosphodonor varies considerably as exemplified by the variability in the levels of phosphorylated RR. SsrB of *Salmonella* gets phosphorylated up to 39%, PrrA of *Rhodobacter* and WalR of *B*. *anthracis* up to 20–25% and WalR of *Streptococcus* up to 95% when incubated with AcP *in vitro* [21, 47–49]. Thus, the paradigm is not as simple as stated and holds a certain degree of diversity when it comes to the affinity of an RR for a particular donor, the extent and rate of autophosphorylation reactions, etc. Our study demonstrates that BAS0540 possess an almost equal affinity for AcP and CP and fits into this classical paradigm. The fact that phosphorylation enhances the DNA binding capability of BAS0540 further amplifies the importance of determining the above parameters for this RR.*In silico* regulon prediction for BAS0540 gave a list of 30 genes, out of which 23 were functionally annotated. DNA binding ability of BAS0540 to 5 regulon candidates was demonstrated *in vitro*. Phosphorylation at the N-terminal of BAS0540 enhances the DNA binding ability of its C-terminal domain, an observation previously drawn for other RRs as well [50, 51]. The fact that the DNA binding capability of BAS0540 is enhanced in the presence of AcP/CP is in perfect sync with the results of *in vitro* phosphorylation of BAS0540 by small molecule phosphodonors. Competition with a molar excess of specific and non-specific probes demonstrates the specificity of BAS0540-DNA interactions.

BAS0540 was one of the candidates in its own putative regulon. We could locate its consensus DNA binding site upstream of the -35 box of its bioinformatically predicted promoter, indicating a positive autoregulation. Autoregulation, which can be either positive or negative is a common attribute of many TCSs and provides the system with certain functional advantages [52].

Another putative candidate in the regulon search was BAS3758, annotated as cell division protein FtsA in *B*. *anthracis*. DOOR database and location of the terminator sequences predicted it to be the first gene of the operon followed by *ftsZ*. BAS0540 binding site was found downstream of the -35 box of an *in silico* predicted promoter for *ftsA* in *B*. *anthracis*, indicative of a negative regulation. In *B*. *subtilis*, it has been reported that since FtsA is recruited in an FtsZ dependent manner to the vegetative and sporulation septa, initiated by the latter itself [53, 54], the ratio of these two proteins is critical for cell division and thus require a tight regulation. Moreover, deletion/downregulation of *ftsA* and/or *ftsZ* causes an impaired cell division, making the cells filamentous during vegetative growth and prevents sporulation [55]. In order to substantiate our observation that BAS0540 regulates *ftsA*, we overexpressed the RR BAS0540 in *B*. *anthracis*. Under the conditions of maximal induction (1mM IPTG), this overexpression resulted in an approximately 6-fold increase in the mean cell length of the bacteria and a 16-fold decrease in its sporulation titer. These phenotypic and physiological alterations can be linked to the negative regulation of *ftsA* and/or *fts*Z. Together, these findings indicate that BAS0540-BAS0541 TCS of *B*. *anthracis* is indeed implicated in cell division and sporulation.

Another candidate, BAS2952, is annotated as heat induced stress protein YflT and is the first gene of a three gene operon in *B*. *anthracis*. In *B*. *subtilis* and *B*. *cereus*, YflT is reported to be induced in response to heat and plays a significant role in the σ^B^–regulated component of the stress response [56–58]. The BAS0540 binding site was found upstream of the -35 box of the bioinformatically predicted promoter for *yflT*, suggesting a positive regulation.

Two other candidates, BAS1041 and BAS5106 are annotated as CAAX amino terminal protease of the Abi superfamily, members of which are involved in conferring immunity to bacteria from the toxic effects of their own bacteriocins [59] and MFP subunit of RND family efflux transporters that consists of nodulation, acriflavin resistance, heavy metal efflux and multidrug resistance proteins respectively. While the BAS0540 binding site was located downstream of the -35 box of a bioinformatically predicted promoter of BAS1041, it was located upstream of the same, for an *in silico* predicted promoter of BAS5106, indicating a negative and positive regulation respectively. TCSs often target genes that encode for transporters, suggesting a coupling between the environmental signals and import/export of compounds.

TCSs contribute a complicated signaling network that might regulate important events of *B*. *anthracis* life cycle, its pathogenesis, and adaptation to the host environment, unraveling of which requires an insightful investigation of these novel TCSs from scratch. Conclusively, our study establishes that BAS0540-BAS0541 is a functional TCS of *B*. *anthracis* and possess the attributes of a prototypical TCS. Regulon prediction and *in vitro* binding assays suggests that this TCS might regulate a diverse set of genes with functions ranging from cell division, sporulation, stress response, cell wall metabolism, general metabolism, antibiotic resistance, and transport. Moreover, the filamentous phenotype and the decrease in sporulation titer obtained upon BAS0540 overexpression in *B*. *anthracis* is indicative that BAS0540-BAS0541 certainly plays a significant role in processes like cell division and sporulation of the pathogen.

## Supporting Information

S1 Fig Operonic organization of BAS0540-BAS0541 of *B*. *anthracis*. (A) cDNA amplification using indicated forward and reverse primers (Red arrows). (B) Genomic organization of BAS0540-BAS0541 operon. There is a 4 nt overlap between the two genes.
(TIF)

S2 Fig Confocal immnofluorescence. (A, B) Cells treated with 1:50 dilution of Pre-immune sera as the primary antibody and 1:100 dilution of secondary antibody only. (C) Z-stack image. Fluorescence due to BAS0540 bound secondary antibody detected only in the middle stacks and not the peripheral stacks.
(TIF)

S3 Fig Phosphotransfer from BAS0541T to BAS0540. (A) Phosphorylated RR was detected as soon as 5 min of [ɣ-^32^ P]ATP addition. (B, C) Intensity analysis was done using ImageJ 1.45S software
(TIF)

S4 Fig Phosphorylation of BAS0540 by AcP and multiple sequence alignment of BAS0540. (A) BAS0540 could be phosphorylated with AcP which increased with time, reaching a maximum of 40–45%. Intensity analysis was done using ImageJ 1.45S software. (B) T-coffee was used to align BAS0540 with its homologs from other Firmicutes. The analysis and visualization was done using Jalview.org.
(TIF)

S5 Fig Functional classification of the candidate genes of the putative regulon of BAS0540 in *B*. *anthracis*. This classification was made by analysis of the constitutive domains of the candidate proteins using Uniprot and NCBI CD search.
(TIF)

S6 Fig Immunoblotting of BAS0540 and BAS0541T using anti-histidine antibody. (A) BAS0540. (B) BAS0541T.
(TIF)

S1 Text *In silico* regulon prediction for BAS0540. Table A depicts functional annotation of putative regulon candidates. Table B contains the results of *in silico regulon* prediction by Regulatory Sequence Analysis Tool (rsat) required for data availability.
(DOCX)

S2 Text Data availability for quantification of gel bands by image j 1.45S software, qRT-PCR, DIC microscopy, spore titer determination and autophosphorylation and phosphotransfer reactions. Table A contains quantification of gel bands for phosphorylation of BAS0540 by CP and AcP. Table B depicts qRT-PCR data. Table C shows mean cell length of wild type *B*. *anthracis* and BAS0540 overexpressed strain from 3 different experiments. Table D depicts average spore titer of wild type *B*. *anthracis* and BAS0540 overexpressed strain from 3 different experiments. Table E depicts intensity units (%) of autophosphorylation and phosphotransfer reactions.
(DOCX)

## Abbreviations

TCS: two-component signal transduction systems
HK: histidine kinase
RR: response regulator
AcP: acetyl phosphate
CP: carbamoyl phosphate
EMSA: electrophoretic mobility shift assay
ELISA: enzyme linked immunosorbent assay
HRP: horseradish peroxidase
AP: alkaline phosphatase
