# Supplementary figures and images for: Identification, Functional Characterization and Regulon Prediction of a Novel Two Component System Comprising BAS0540-BAS0541 of Bacillus anthracis
Source: PLoS One. 2016 Jul 8;11(7):e0158895. doi: 10.1371/journal.pone.0158895 (PMC4938410; doi:10.1371/journal.pone.0158895)

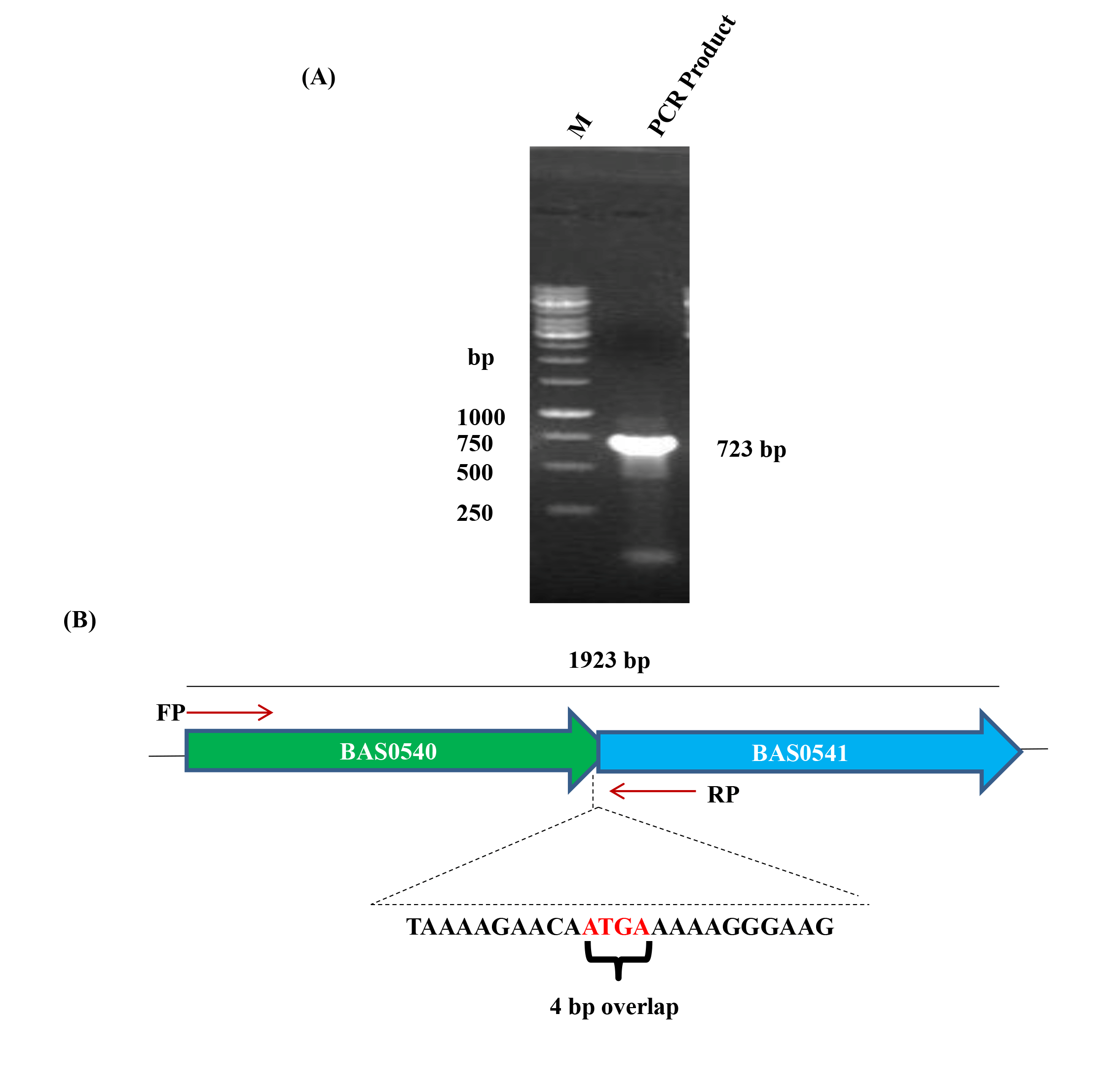

Supplement: S1 Fig — (A) cDNA amplification using indicated forward and reverse primers (Red arrows). (B) Genomic organization of BAS0540-BAS0541 operon. There is a 4 nt overlap between the two genes. (TIF) [file pone.0158895.s001.tif]

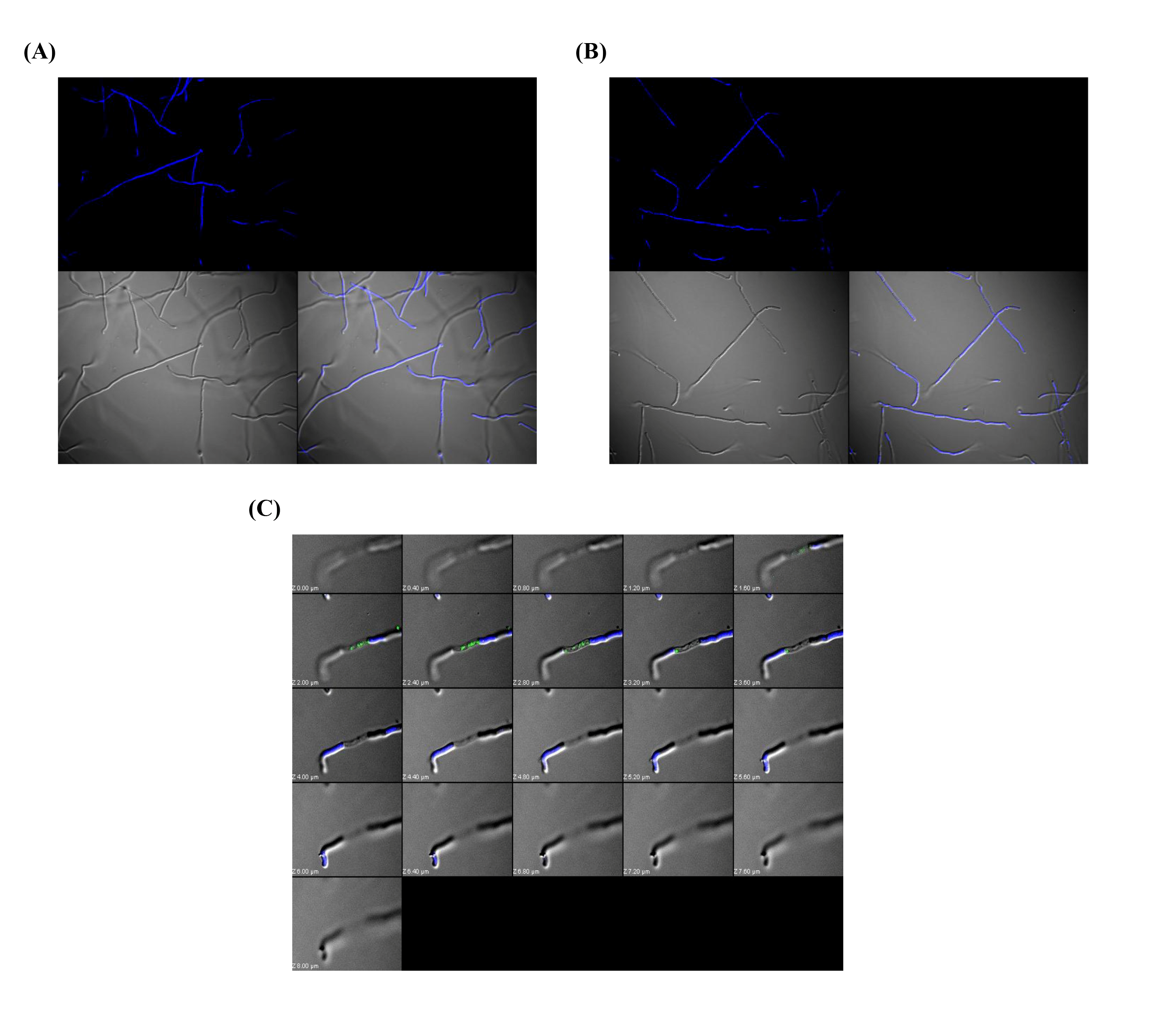

Supplement: S2 Fig — (A, B) Cells treated with 1:50 dilution of Pre-immune sera as the primary antibody and 1:100 dilution of secondary antibody only. (C) Z-stack image. Fluorescence due to BAS0540 bound secondary antibody detected only in the middle stacks and not the peripheral stacks. (TIF) [file pone.0158895.s002.tif]

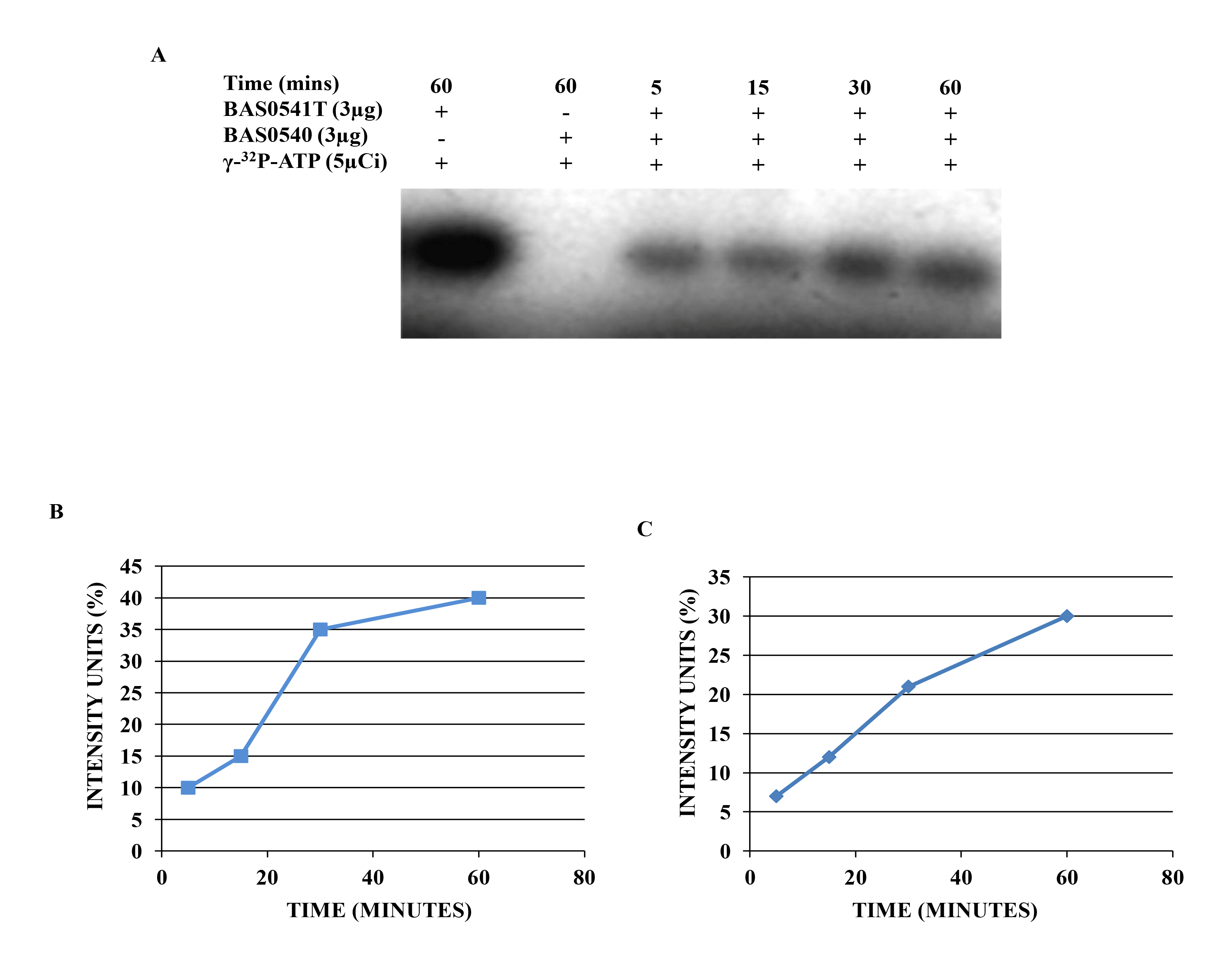

Supplement: S3 Fig — (A) Phosphorylated RR was detected as soon as 5 min of [ɣ-32 P]ATP addition. (B, C) Intensity analysis was done using ImageJ 1.45S software (TIF) [file pone.0158895.s003.tif]

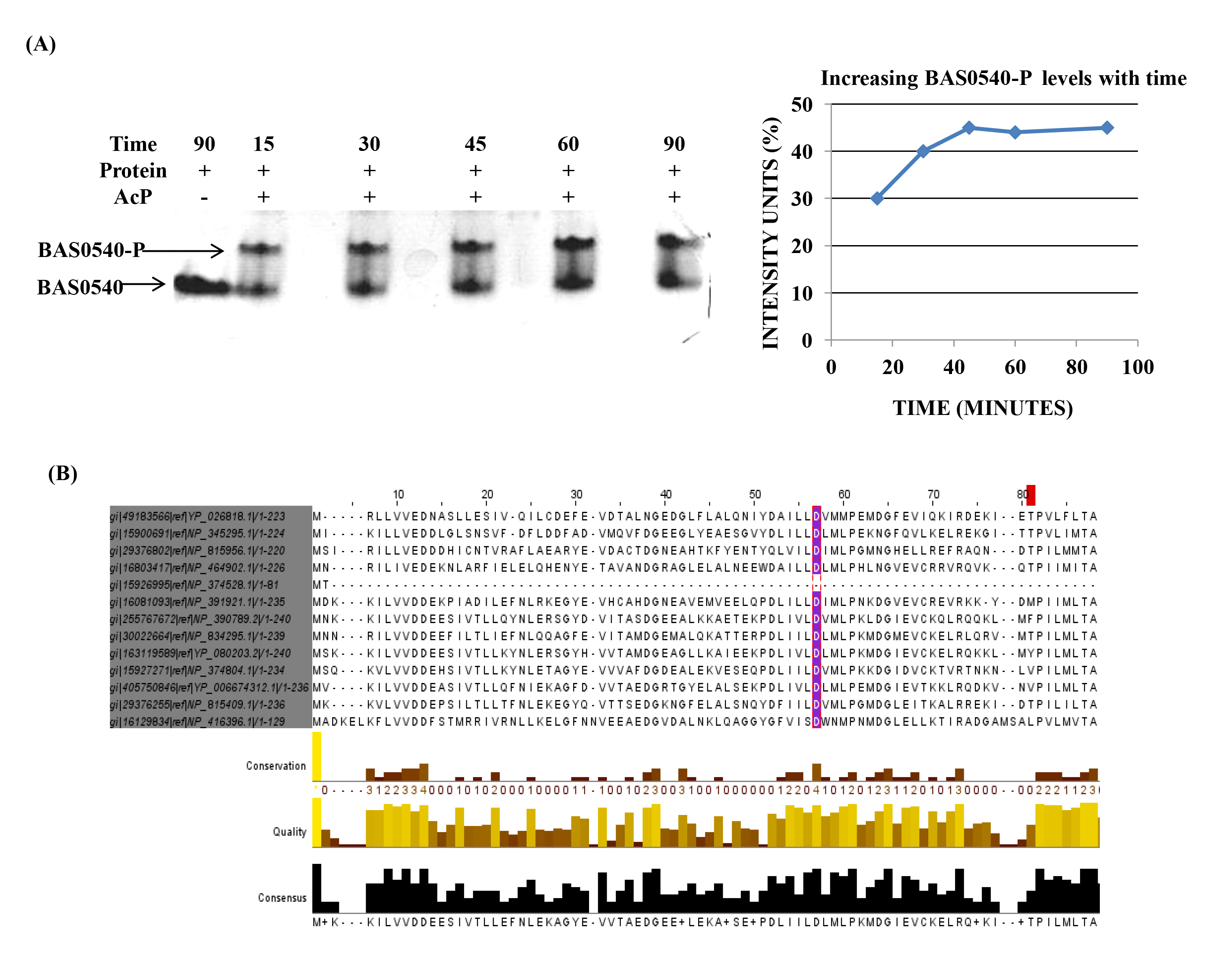

Supplement: S4 Fig — (A) BAS0540 could be phosphorylated with AcP which increased with time, reaching a maximum of 40–45%. Intensity analysis was done using ImageJ 1.45S software. (B) T-coffee was used to align BAS0540 with its homologs from other Firmicutes. The analysis and visualization was done using Jalview.org. (TIF) [file pone.0158895.s004.tif]

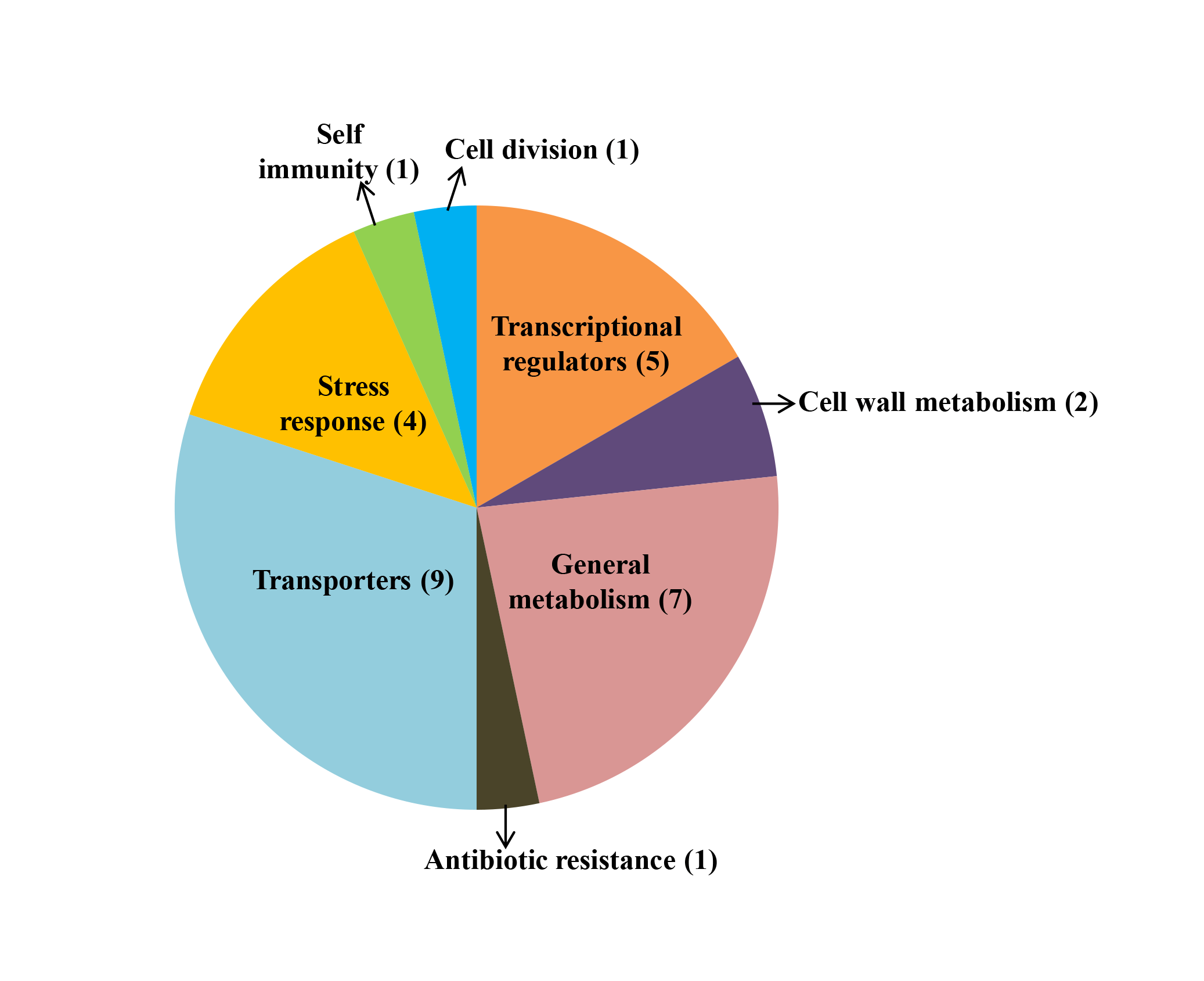

Supplement: S5 Fig — This classification was made by analysis of the constitutive domains of the candidate proteins using Uniprot and NCBI CD search. (TIF) [file pone.0158895.s005.tif]

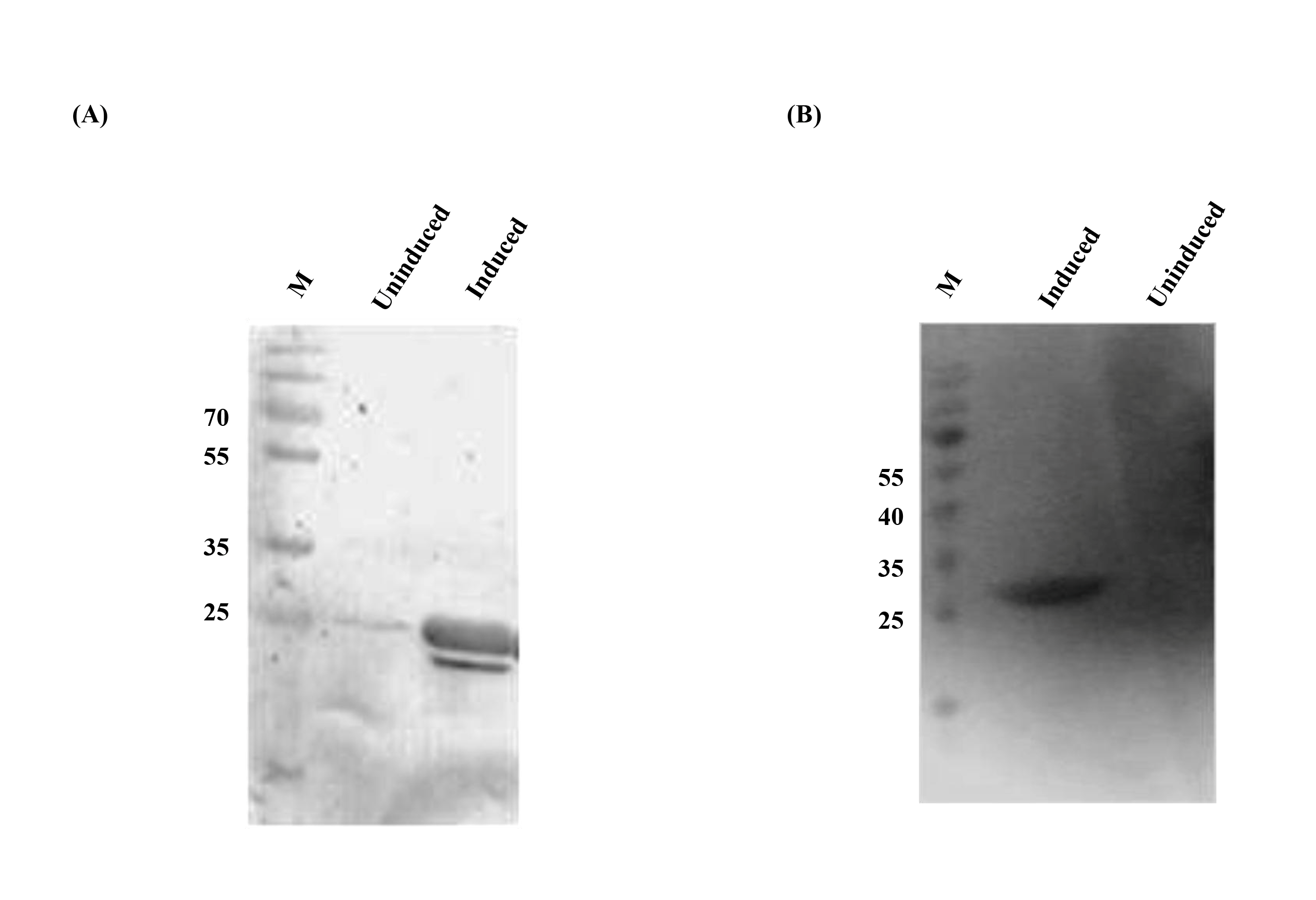

Supplement: S6 Fig — (A) BAS0540. (B) BAS0541T. (TIF) [file pone.0158895.s006.tif]
