# Supplementary material for: Identification, Functional Characterization and Regulon Prediction of a Novel Two Component System Comprising BAS0540-BAS0541 of Bacillus anthracis
Source: PLoS One. 2016 Jul 8;11(7):e0158895. doi: 10.1371/journal.pone.0158895 (PMC4938410; doi:10.1371/journal.pone.0158895)
Supplement: S1 Text — Table A depicts functional annotation of putative regulon candidates. Table B contains the results of in silico regulon prediction by Regulatory Sequence Analysis Tool (rsat) required for data availability. (DOCX) [file pone.0158895.s007.docx]

**Supporting Text 1 (S1 text)**

**Table A**

Putative regulon of BAS0540 in *B. anthracis*

|  | Gene (Protein) | Protein name | Functional Prediction/ Conserved domain/ superfamily CDD search/ litreature |
| --- | --- | --- | --- |
| 1 | BAS0540 ([YP_026818](http://www.ncbi.nlm.nih.gov/protein/YP_026818)) | DNA binding response regulator | OmpR family RR [COG0745](http://www.ncbi.nlm.nih.gov/Structure/cdd/cddsrv.cgi?ascbin=8&maxaln=10&seltype=2&uid=COG0745) |
| 2 | BAS0483 (YP_026763) | Diacylglycerol glucosyltransferase | Cell membrane, lipoteichoic acid, peptidoglycan synthesis [PRK13609](http://www.ncbi.nlm.nih.gov/Structure/cdd/cddsrv.cgi?ascbin=8&maxaln=10&seltype=2&uid=PRK13609) |
| 3 | BAS0527 ([YP_026805](http://www.ncbi.nlm.nih.gov/protein/YP_026805)) | CitB family, sensor kinase | Sensing C4-dicarboxylates which after sugars are one of the most important and preferred substrates for bacteria [COG3290](http://www.ncbi.nlm.nih.gov/Structure/cdd/cddsrv.cgi?ascbin=8&maxaln=10&seltype=2&uid=COG3290) |
| 4 | BAS0647 ([YP_026924](http://www.ncbi.nlm.nih.gov/protein/YP_026924)) | VanZ family protein | Putative cell wall related functions. Confers low level resistance to glycopeptide antibiotics (cell wall synthesis inhibition) by an unknown mechanism [pfam04892](http://www.ncbi.nlm.nih.gov/Structure/cdd/cddsrv.cgi?ascbin=8&maxaln=10&seltype=2&uid=pfam04892) |
| 5 | BAS0680 (YP_026957) | Ferrous iron transport protein A | FeoA superfamily; This family includes FeoA, a small protein, probably involved in Fe^2+^ transport. [pfam04023](http://www.ncbi.nlm.nih.gov/Structure/cdd/cddsrv.cgi?ascbin=8&maxaln=10&seltype=2&uid=pfam04023) |
| 6 | BAS0681 ([YP_026958](http://www.ncbi.nlm.nih.gov/protein/YP_026958)) | Phosphate ABC transporter substrate-binding protein | Substrate binding domain of putative ABC-type phosphate transporter. Contains uncharacterized phosphate binding domains found in PstS proteins that serve as initial receptors in the ABC transport of phosphate in eubacteria and archaea.  [cd13653](http://www.ncbi.nlm.nih.gov/Structure/cdd/cddsrv.cgi?ascbin=8&maxaln=10&seltype=2&uid=cd13653) |
| 7 | BAS0875 ([YP_027151](http://www.ncbi.nlm.nih.gov/protein/YP_027151)) | MerR family transcriptional regulator | Helix-Turn-Helix DNA binding domain of transcription regulators from the MerR superfamily that have been shown to mediate responses to stress including exposure to heavy metals, drugs, or oxygen radicals in eubacterial and some archaeal species [COG0789](http://www.ncbi.nlm.nih.gov/Structure/cdd/cddsrv.cgi?ascbin=8&maxaln=10&seltype=2&uid=COG0789) |
| 8 | BAS1040 (YP_027312) | DNA-binding response regulator | OmpR family RR [COG0745](http://www.ncbi.nlm.nih.gov/Structure/cdd/cddsrv.cgi?ascbin=8&maxaln=10&seltype=2&uid=COG0745) |
| 9 | BAS1041 (YP_027313) | CAAX amino terminal protease family protein | Abi superfamily; CAAX protease self-immunity [pfam02517](http://www.ncbi.nlm.nih.gov/Structure/cdd/cddsrv.cgi?ascbin=8&maxaln=10&seltype=2&uid=pfam02517) |
| 10 | BAS1732 ([YP_027997](http://www.ncbi.nlm.nih.gov/protein/YP_027997)) | Oligopeptide ABC transporter solute binding protein | The substrate-binding component of an ABC-type oligopetide import system [COG4166](http://www.ncbi.nlm.nih.gov/Structure/cdd/cddsrv.cgi?ascbin=8&maxaln=10&seltype=2&uid=COG4166) |
| 11 | BAS2226 (YP_028487) | alpha/beta hydrolase | [COG0596](http://www.ncbi.nlm.nih.gov/Structure/cdd/cddsrv.cgi?ascbin=8&maxaln=10&seltype=2&uid=COG0596) |
| 12 | BAS2257 (YP_028518) | cold shock protein CspA | Cold-Shock Protein (CSP) contains an S1-like cold-shock domain (CSD) that is found in eukaryotes, prokaryotes, and archaea [cd04458](http://www.ncbi.nlm.nih.gov/Structure/cdd/cddsrv.cgi?ascbin=8&maxaln=10&seltype=2&uid=cd04458) |
| 13 | BAS2414  (YP_028674) | Mur ligase family protein | This family contains a number of related ligase enzymes. This family includes: MurC, MurD, MurE, MurF, Mpl and FolC. MurC, MurD, MurE and MurF catalyse consecutive steps in the synthesis of peptidoglycan pfam01225 |
| 14 | BAS2952 (YP_029209) | Heat induced stress protein YflT | YflT is a heat induced protein [pfam11181](http://www.ncbi.nlm.nih.gov/Structure/cdd/cddsrv.cgi?ascbin=8&maxaln=10&seltype=2&uid=pfam11181) |
| 15 | BAS3644 (YP_029895) | GntR family transcriptional regulator | Winged helix-turn-helix (WHTH) DNA-binding domain of the GntR family of transcriptional regulators [COG2188](http://www.ncbi.nlm.nih.gov/Structure/cdd/cddsrv.cgi?ascbin=8&maxaln=10&seltype=2&uid=COG2188) |
| 16 | BAS3758 (YP_030009) | Cell division protein FtsA | Cell division ATPase FtsA [Cell cycle control, cell division, chromosome partitioning] [COG0849](http://www.ncbi.nlm.nih.gov/Structure/cdd/cddsrv.cgi?ascbin=8&maxaln=10&seltype=2&uid=COG0849) |
| 17 | BAS3874 (YP_030124) | Lysine decarboxylase | Amino acid transport and metabolism [COG1982](http://www.ncbi.nlm.nih.gov/Structure/cdd/cddsrv.cgi?ascbin=8&maxaln=10&seltype=2&uid=COG1982) |
| 18 | BAS3885 ([YP_030135](http://www.ncbi.nlm.nih.gov/protein/YP_030135)) | HAD superfamily hydrolase | [Metabolism and transport COG0561](http://www.ncbi.nlm.nih.gov/Structure/cdd/cddsrv.cgi?ascbin=8&maxaln=10&seltype=2&uid=COG0561) |
| 19 | BAS3918 ([YP_030168](http://www.ncbi.nlm.nih.gov/protein/YP_030168)) | aminotransferase | Amino acid metabolism [PRK07683](http://www.ncbi.nlm.nih.gov/Structure/cdd/cddsrv.cgi?ascbin=8&maxaln=10&seltype=2&uid=PRK07683) |
| 20 | BAS4912 (YP_031153) | TrkA domain-containing protein | K+/H+ antiporter YhaU, regulatory subunit KhtT [Inorganic ion transport and metabolism]  [COG0490](http://www.ncbi.nlm.nih.gov/Structure/cdd/cddsrv.cgi?ascbin=8&maxaln=10&seltype=2&uid=COG0490) |
| 21 | BAS4913 (YP_031154) | Sodium/alanine symporter family protein | Na+/alanine symporter [Amino acid transport and metabolism [COG1115](http://www.ncbi.nlm.nih.gov/Structure/cdd/cddsrv.cgi?ascbin=8&maxaln=10&seltype=2&uid=COG1115) |
| 22 | BAS5106 (YP_031345) | RND family efflux transporter MFP subunit | This model represents the MFP (membrane fusion protein) component of the RND family of transporters. RND refers to Resistance, Nodulation, and cell Division [TIGR01730](http://www.ncbi.nlm.nih.gov/Structure/cdd/cddsrv.cgi?ascbin=8&maxaln=10&seltype=2&uid=TIGR01730) |
| 23 | BAS5107 ([YP_031346](http://www.ncbi.nlm.nih.gov/protein/YP_031346)) | DNA-binding protein | Helix-turn-helix XRE-family like proteins; prokaryotic DNA binding proteins belonging to the xenobiotic response element family of transcriptional regulators cd0093 |

**Table B (data availability)**

*In silico* regulon prediction using Regulatory Sequence Analysis Tool (rsat)

(Note-The table does not include hypothetical genes)

Input format fasta

Pattern file $RSAT/public_html/tmp/apache/2016/03/22/gs-dna-pattern_2016-03-22.020638_vEybto.pat

Search method IUPAC

Threshold 0

Allowed substitutions 1

Return fields

sites

Patterns

seq id score

NTTAAGNNNNNTTTAAG NTTAAGNNNNNTTTAAG 1

Matching positions

| PatID | Strand | Pattern | SeqID | Start | End | matching_seq | Score |
| --- | --- | --- | --- | --- | --- | --- | --- |
| NTTAAGNNN NNTTTAAG | D | NTTAAGNNNNNTTTAAG | YP_026763.1\|Bacillus_anthracis_Sterne_uid58091\|BAS0483 | -81 | -65 | TTTATGTAATTTTTAAG | 0.94 |
| NTTAAGNNNNNTTTAAG | D | NTTAAGNNNNNTTTAAG | YP_026805.1\|Bacillus_anthracis_Sterne_uid58091\|BAS0527 | -61 | -45 | ATTAAGTATAATTGAAG | 0.94 |
| NTTAAGNNNNNTTTAAG | D | NTTAAGNNNNNTTTAAG | YP_026818.1\|Bacillus_anthracis_Sterne_uid58091\|BAS0540 | -113 | -97 | ATTAAGCAAAATTTAAG | 1 |
| NTTAAGNNNNNTTTAAG | D | NTTAAGNNNNNTTTAAG | YP_026924.1\|Bacillus_anthracis_Sterne_uid58091\|BAS0647 | -69 | -53 | TTTAATTTTGTTTTAAG | 0.94 |
| NTTAAGNNNNNTTTAAG | D | NTTAAGNNNNNTTTAAG | YP_026957.1\|Bacillus_anthracis_Sterne_uid58091\|BAS0680 | -422 | -406 | GTTAAGCAATTTTTAAT | 0.94 |
| NTTAAGNNNNNTTTAAG | R | NTTAAGNNNNNTTTAAG | YP_026958.1\|Bacillus_anthracis_Sterne_uid58091\|BAS0681 | -80 | -64 | GTTAAGCAATTTTTAAT | 0.94 |
| NTTAAGNNNNNTTTAAG | D | NTTAAGNNNNNTTTAAG | YP_027151.1\|Bacillus_anthracis_Sterne_uid58091\|BAS0875 | -325 | -309 | ATTAAGTAATGATTAAG | 0.94 |
| NTTAAGNNNNNTTTAAG | R | NTTAAGNNNNNTTTAAG | YP_027312.1\|Bacillus_anthracis_Sterne_uid58091\|BAS1040 | -75 | -59 | TTTAAGGAAATTTTAAG | 1 |
| NTTAAGNNNNNTTTAAG | D | NTTAAGNNNNNTTTAAG | YP_027313.1\|Bacillus_anthracis_Sterne_uid58091\|BAS1041 | -88 | -72 | CTTATGGAATTTTTAAG | 0.94 |
| NTTAAGNNNNNTTTAAG | R | NTTAAGNNNNNTTTAAG | YP_027997.1\|Bacillus_anthracis_Sterne_uid58091\|BAS1732 | -380 | -364 | CTTAAGGATTTTGTAAG | 0.94 |
| NTTAAGNNNNNTTTAAG | D | NTTAAGNNNNNTTTAAG | YP_028487.1\|Bacillus_anthracis_Sterne_uid58091\|BAS2226 | -178 | -162 | ATTAAGAAAATATTAAG | 0.94 |
| NTTAAGNNNNNTTTAAG | D | NTTAAGNNNNNTTTAAG | YP_028518.1\|Bacillus_anthracis_Sterne_uid58091\|BAS2257 | -161 | -145 | CTTAAGGTCAATTTAAC | 0.94 |
| NTTAAGNNNNNTTTAAG | R | NTTAAGNNNNNTTTAAG | YP_028674.1\|Bacillus_anthracis_Sterne_uid58091\|BAS2414 | -185 | -169 | GTTAAGGTTACTTAAAG | 0.94 |
| NTTAAGNNNNNTTTAAG | R | NTTAAGNNNNNTTTAAG | YP_029209.1\|Bacillus_anthracis_Sterne_uid58091\|BAS2952 | -368 | -352 | ATTAAGAAAACTTTAAG | 1 |
| NTTAAGNNNNNTTTAAG | R | NTTAAGNNNNNTTTAAG | YP_029895.1\|Bacillus_anthracis_Sterne_uid58091\|BAS3644 | -136 | -120 | ATTAATAATTTTTTAAG | 0.94 |
| NTTAAGNNNNNTTTAAG | R | NTTAAGNNNNNTTTAAG | YP_030009.1\|Bacillus_anthracis_Sterne_uid58091\|BAS3758 | -154 | -138 | TTTAACATTCATTTAAG | 0.94 |
| NTTAAGNNNNNTTTAAG | R | NTTAAGNNNNNTTTAAG | YP_030124.1\|Bacillus_anthracis_Sterne_uid58091\|BAS3874 | -73 | -57 | GTTAAGTTCTTTTTTAG | 0.94 |
| NTTAAGNNNNNTTTAAG | R | NTTAAGNNNNNTTTAAG | YP_030135.1\|Bacillus_anthracis_Sterne_uid58091\|BAS3885 | -398 | -382 | CTTAAGCGTTATTTAAA | 0.94 |
| NTTAAGNNNNNTTTAAG | R | NTTAAGNNNNNTTTAAG | YP_030168.1\|Bacillus_anthracis_Sterne_uid58091\|BAS3918 | -43 | -27 | ATTAAGTTCATTTTAAC | 0.94 |
| NTTAAGNNNNNTTTAAG | D | NTTAAGNNNNNTTTAAG | YP_031153.1\|Bacillus_anthracis_Sterne_uid58091\|BAS4912 | -153 | -137 | TTTAAGTATCTTTTTAG | 0.94 |
| NTTAAGNNNNNTTTAAG | R | NTTAAGNNNNNTTTAAG | YP_031154.1\|Bacillus_anthracis_Sterne_uid58091\|BAS4913 | -275 | -259 | TTTAAGTATCTTTTTAG | 0.94 |
| NTTAAGNNNNNTTTAAG | R | NTTAAGNNNNNTTTAAG | YP_031345.1\|Bacillus_anthracis_Sterne_uid58091\|BAS5106 | -173 | -157 | TTTAAGATAGATTTAAG | 1 |
| NTTAAGNNNNNTTTAAG | D | NTTAAGNNNNNTTTAAG | YP_031346.1\|Bacillus_anthracis_Sterne_uid58091\|BAS5107 | -209 | -193 | TTTAAGATAGATTTAAG | 1 |
