# Supplementary material for: Identification, Functional Characterization and Regulon Prediction of a Novel Two Component System Comprising BAS0540-BAS0541 of Bacillus anthracis
Source: PLoS One. 2016 Jul 8;11(7):e0158895. doi: 10.1371/journal.pone.0158895 (PMC4938410; doi:10.1371/journal.pone.0158895)
Supplement: S2 Text — Table A contains quantification of gel bands for phosphorylation of BAS0540 by CP and AcP. Table B depicts qRT-PCR data. Table C shows mean cell length of wild type B. anthracis and BAS0540 overexpressed strain from 3 different experiments. Table D depicts average spore titer of wild type B. anthracis and BAS0540 overexpressed strain from 3 different experiments. Table E depicts intensity units (%) of autophosphorylation and phosphotransfer reactions. (DOCX) [file pone.0158895.s008.docx]

**Supporting text 2 (S2 text)**

**Table A**

**Data availability for quantification of gel bands by image j 1.45S software**

BAS0540 phosphorylation by carbamoyl phosphate

| Time | Experiment 1 | Area | % |  |  | Experiment 2 | Area | % |
| --- | --- | --- | --- | --- | --- | --- | --- | --- |
|  |  |  |  |  |  |  |  |  |
| 15 minutes | Only protein | 45902.16 | 67 |  |  | Only protein | 38933.67 | 66.15 |
|  | BAS0540-P | 22608.52 | 33 |  |  | BAS0540-P | 19046.47 | 33.85 |
|  |  |  |  |  |  |  |  |  |
|  |  |  |  |  |  |  |  |  |
| 30 minutes | Only protein | 44260.84 | 64.604 |  |  | Only protein | 39122.66 | 63.195 |
|  | BAS0540-P | 24249.84 | 35.396 |  |  | BAS0540-P | 22785.08 | 36.805 |
|  |  |  |  |  |  |  |  |  |
|  |  |  |  |  |  |  |  |  |
| 45 minutes | Only protein | 43611.32 | 59.327 |  |  | Only protein | 45819.71 | 58.202 |
|  | BAS0540-P | 29898.86 | 40.673 |  |  | BAS0540-P | 32906.05 | 41.798 |
|  |  |  |  |  |  |  |  |  |
|  |  |  |  |  |  |  |  |  |
| 60 minutes | Only protein | 40608.64 | 56 |  |  | Only protein | 56548.19 | 56.328 |
|  | BAS0540-P | 31906.79 | 44 |  |  | BAS0540-P | 43842.71 | 43.672 |
|  |  |  |  |  |  |  |  |  |
|  |  |  |  |  |  |  |  |  |
| 90 minutes | Only protein | 53928.1 | 55.19 |  |  | Only protein | 39883.48 | 54.99 |
|  | BAS0540-P | 43767.74 | 44.8 |  |  | BAS0540-P | 32631.94 | 45 |

BAS0540 phosphorylation by acetyl phosphate

| Time | Experiment 1 | Area | % |  | Experiment 2 | Area | % |
| --- | --- | --- | --- | --- | --- | --- | --- |
|  |  |  |  |  |  |  |  |
| 15 minutes | Only protein | 44531.94 | 70 |  | Only protein | 47957.476 | 70 |
|  | BAS0540-P | 23978.73 | 30 |  | BAS0540-P | 20553.204 | 30 |
|  |  |  |  |  |  |  |  |
|  |  |  |  |  |  |  |  |
| 30 minutes | Only protein | 34904.59 | 60.2 |  | Only protein | 40914.578 | 59.72 |
|  | BAS0540-P | 23076.45 | 39.8 |  | BAS0540-P | 27596.102 | 40.28 |
|  |  |  |  |  |  |  |  |
|  |  |  |  |  |  |  |  |
| 45 minutes | Only protein | 35460.39 | 55.38 |  | Only protein | 32203.12 | 54.7 |
|  | BAS0540-P | 28570.65 | 44.62 |  | BAS0540-P | 26669.13 | 45.3 |
|  |  |  |  |  |  |  |  |
|  |  |  |  |  |  |  |  |
| 60 minutes | Only protein | 34668.33 | 56 |  | Only protein | 40825.472 | 56.299 |
|  | BAS0540-P | 27239.41 | 44 |  | BAS0540-P | 31689.957 | 43.701 |
|  |  |  |  |  |  |  |  |
|  |  |  |  |  |  |  |  |
| 90 minutes | Only protein | 33064.21 | 54.8 |  | Only protein | 39956.002 | 55.1 |
|  | BAS0540-P | 30966.83 | 45.2 |  | BAS0540-P | 32559.428 | 44.9 |

**Table B**

**Data availability for qRT-PCR.**

The normalization was done by calculating the difference between Ct of endogenous control and BAS0540. The values used to plot the graph are provided, excluding the raw data.

| O.D _600nm_ | Normalized Ct values (Average of two experiments) |
| --- | --- |
| 0.3 | 3.8 |
| 0.6 | 4.2 |
| 0.9 | 3.9 |
| 1.2 | 4 |

**Table C**

**Data availability for cell length measurement by DIC microscopy**

Mean cell length of 10 cells from three different experiments was calculated. SD is also depicted in the table.

| Strain | Mean cell length | SD |
| --- | --- | --- |
| Wild type *B. anthracis* | 4.18 | 0.7 |
| BAS0540 overexpressed *B. anthracis* | 24.52 | 4.26 |

**Table D**

**Data availability for sporulation titer of wild type *B. anthracis* and BAS0540 overexpressed strain**

Average spore titer calculated from three independent experiments is depicted in the table.

| Strain | Average spore titer |
| --- | --- |
| Wild type *B. anthracis* | 3.45 X 10^6^ |
| BAS0540 overexpressed *B. anthracis* | 2.1 X 10^5^ |

**Table E**

**Data availability for quantification of gel bands in autophosphorylation reaction of kinase BAS0541T and phosphotransfer reaction between BAS0541T and BAS0540**

| Autophosphorylation of BAS0541T | | BAS0541T-BAS0540 Phosphotransfer | |
| --- | --- | --- | --- |
| Time (minutes) | **Intensity units (%)** | **Time (minutes)** | **Intensity units (%)** |
| 5 | 10 | 5 | 7 |
| 15 | 15 | 15 | 12 |
| 30 | 35 | 30 | 21 |
| 60 | 40 | 60 | 30 |
